# Unlocking New Pharma/Nutraceutical Frontiers With Neuroprotective Properties of Three *Hypericum* Species: A Study Combination With In Vitro and In Silico Methodologies

**DOI:** 10.1002/fsn3.70069

**Published:** 2025-04-10

**Authors:** Muammer Bahsi, Simonetta Cristina Di Simone, Dimitrina Zheleva‐Dimitrova, Gokhan Zengin, Gaia Cusumano, Giancarlo Angeles Flores, Paola Angelini, Carla Emiliani, Mehmet Veysi Cetiz, Annalisa Chiavaroli, Luigi Menghini, Guistino Orlando, Claudio Ferrante

**Affiliations:** ^1^ Faculty of Education Firat University Elazığ Turkey; ^2^ Botanic Garden “Giardino Dei Semplici”, Department of Pharmacy “Gabriele d'Annunzio” University Chieti Italy; ^3^ Department of Pharmacognosy, Faculty of Pharmacy Medical University of Sofia Sofia Bulgaria; ^4^ Department of Biology, Science Faculty Selcuk University Konya Turkey; ^5^ Department of Chemistry, Biology and Biotechnology University of Perugia Perugia Italy; ^6^ Department of Medical Biochemistry, Faculty of Medicine Harran University Sanliurfa Turkey

**Keywords:** antioxidant, *Hypericum*, hyperoside, in silico methods, neuromodulator, novel pharmaceutics

## Abstract

This study investigates the phytochemical composition and biopharmacological potential of three *Hypericum* species ( *H. scabrum* , *H. lysimachioides*, and *H. uniglandulosum*) from Turkey. Aqueous and hydroalcoholic extracts were analyzed for their total phenolic content (TPC), total flavonoid content (TFC), and individual components (by the UHPLC–HRMS technique). Antioxidant activities were investigated by DPPH, ABTS, CUPRAC, FRAP, phosphomolybdenum, and metal chelating assays. The inhibition effects of the tested extracts on acetylcholinesterase (AChE), butyrylcholinesterase (BChE), tyrosinase, amylase, and glucosidase were examined. One hundred compounds were identified in the chemical composition, and specific compounds for the genus *Hypericum*, such as hyperoside, hypericin, and pseudohypericin, were detected. The highest TPC was detected in the ethanol/water extract of *H. lysimachioides* with 69.21 mg GAE/g. Furthermore, the ethanol/water extract showed the strongest free radical and reducing effect. The ethanol/water extracts of the tested *Hypericum* species were more active in tyrosinase, amylase, and glucosidase than the water extracts. Neuroprotective assessments indicated downregulation of COX‐2 and NOS‐2 genes in LPS‐stimulated mouse cortex models, alongside modulation of SERT and NET expression, suggesting reduced neuroinflammation and enhanced neurotransmitter release. Molecular docking and dynamics analyses highlighted strong binding interactions, especially in the NET_hyperoside and NET_myricitrin complexes. The results indicate significant therapeutic potential for these extracts, supporting their development as natural agents against oxidative stress, neuroinflammation, and related neurodegenerative diseases.

## Graphical abstract

This study investigates the phytochemical composition and biopharmacological potential of the extracts from three *Hypericum* species ( *H. scabrum* , *H. lysimachioides*, and *H. uniglandulosum*) from Turkey. The results indicate significant therapeutic potential for these extracts, supporting their development as natural agents against oxidative stress, neuroinflammation, and related neurodegenerative diseases.

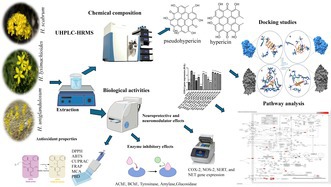

## 1 Introduction

Medicinal plants represent a fundamental resource for the prevention and treatment of numerous diseases, thanks to their antioxidant properties. Plant‐derived antioxidants play a crucial role in reducing oxidative stress, a process closely linked to the pathogenesis of various chronic diseases, including cardiovascular, neurodegenerative, and oncological conditions (Kasote et al. 2015). Phytochemicals such as polyphenols and vitamins can modulate the accumulation of reactive oxygen species (ROS), providing significant protection against oxidative damage at the cellular level (Lee and Park 2021). Plants possess both enzymatic and nonenzymatic antioxidant defense mechanisms. Antioxidant enzymes, such as superoxide dismutase (SOD), catalase (CAT), and glutathione peroxidase (GPx), act by neutralizing free radicals, while nonenzymatic compounds, including ascorbic acid and flavonoids, effectively contribute to mitigating oxidative stress (Kasote et al. 2015). The ability to produce such antioxidants in response to environmental stress represents a key aspect of the phytotherapeutic properties of plants. Recent research has focused on the therapeutic application of plant‐derived antioxidants for the treatment of degenerative and inflammatory diseases (Tyler and Tyler 2023).

In this context, species of the *Hypericum* genus, known for their biological properties, represent a significant example of how medicinal plants can be used to develop innovative therapeutic approaches that are more effective and have fewer adverse effects. The genus *Hypericum*, belonging to the Hypericaceae family, comprises over 500 species distributed worldwide, with a particular concentration in temperate, subtropical, and tropical mountainous regions (Crockett and Robson 2011; Robson 2010). This genus has attracted significant scientific interest due to its numerous medicinal properties and the therapeutic potential demonstrated by various species, traditionally used to treat a wide range of ailments (Far et al. 2024). *Hypericum* species are widely distributed and adapt to different types of habitats and climatic conditions, ranging from hilly regions to humid and arid areas, demonstrating a remarkable capacity for adaptation and resilience. The species of the genus *Hypericum* are known for a variety of biological activities, including antioxidant, antimicrobial, anti‐inflammatory, and wound‐healing effects (Barnes et al. 2001; Whiskey et al. 2001; Zirak et al. 2019). These properties are attributed to a broad spectrum of secondary metabolites, such as flavonoids, acylphloroglucinols, and naphthodianthrones, which confer significant therapeutic versatility to these plants. The flavonoids present in *Hypericum* species have demonstrated strong antioxidant activity, making them useful in combating oxidative stress and reducing cellular damage (Kakouri et al. 2023). Additionally, acylphloroglucinols, such as hyperforin, possess potent antimicrobial and anti‐inflammatory effects, proving effective in treating infections and chronic inflammation (Avato 2005; Ramos‐Hryb et al. 2018). Naphthodianthrones, including hypericin, are known for their antiviral and antidepressant properties, making these plants an interesting option for managing mood disorders and viral infections (Far et al. 2024). Numerous studies have highlighted the potential of these species not only as phytotherapeutic agents for common diseases but also as sources of new bioactive compounds for the development of innovative therapeutic approaches (Marrelli et al. 2020). *Hypericum* species have been studied for their potential in treating neurodegenerative disorders, such as Alzheimer's and Parkinson's diseases, due to their ability to modulate neuroinflammation and protect nerve cells from oxidative stress (Suryawanshi et al. 2024). Additionally, the wound‐healing activity of *Hypericum* has been the subject of in‐depth research, showing promising results in promoting tissue regeneration and accelerating wound healing (Altıparmak et al. 2019). From a phytochemical perspective, *Hypericum* species are characterized by a wide variety of chemical compounds, including hypericin, hyperforin, pseudohypericin, rutin, quercetin (Marrelli et al. 2014), xanthones, tannins, proanthocyanidins, and catechins, contributing to the pharmacological properties of these plants (Alahmad et al. 2022; Çirak et al. 2009). The chemical diversity of these species allows for a wide range of therapeutic applications, making them promising for the discovery of new drugs. In addition, essential oils extracted from *Hypericum* plants possess antimicrobial properties that can be exploited for treating skin infections, while tannins contribute to astringent and anti‐inflammatory activities (Far et al. 2024). Among the species of the genus *Hypericum*, *Hypericum perforatum* is the most studied species; however, other species, including *Hypericum scabrum*, *Hypericum lysimachioides*, and *Hypericum uniglandulosum*, also exhibit medicinal properties and will be further investigated in this study. *H. scabrum* is known for its pharmacological properties, including antidepressant, antioxidant, and anticonvulsant effects. Studies have shown that extracts from this species exhibit protective activity against hypoxia and moderate free radical scavenging capacity, suggesting its possible use in managing oxidative stress (Eslami et al. 2011). Additionally, the antimicrobial and antifungal activities observed in *H. scabrum* are attributed to its essential oils and the presence of omega‐3 and omega‐6 fatty acids, which play an important role in its chemical profile (Shafaghat 2011). These properties make *H. scabrum* a promising option for the development of natural therapies aimed at addressing infections and oxidative stress‐related conditions. *H. lysimachioides* has been studied for its potential benefits in regulating lipid metabolism, particularly by improving lipid profiles and reducing LDL cholesterol levels. The ethanolic extract of this species has shown antioxidant activity comparable to that of vitamin E, suggesting its possible role in preventing cardiovascular diseases (Hakimoğlu et al. 2007). In addition, the essential oil of *H. lysimachioides* contains bioactive compounds with antimicrobial properties, making it useful for treating bacterial infections (Toker et al. 2006). *H. uniglandulosum*, a species endemic to Turkey, is notable for its high concentration of phenolic compounds, such as hypericin and hyperforin, which are largely responsible for its antimicrobial and antioxidant properties (Turkoglu et al. 2015). Research has shown that the essential oil of this species is rich in α‐pinene, a component that contributes to its biological activity, particularly its antimicrobial effects (Babacan and Bagci 2017). These characteristics make *H. uniglandulosum* worthy of further investigation, with potential applications in developing natural antibacterial agents. Overall, these three species of *Hypericum*— *H. scabrum* , *H. lysimachioides*, and *H. uniglandulosum*—demonstrate a range of pharmacological activities that could be useful for therapeutic purposes. Their bioactive compounds and observed biological activities suggest that these species may contribute to the development of natural treatments, offering potential alternatives to conventional synthetic drugs with fewer side effects.

The aim of the present study was to investigate the phytochemical and biopharmacological properties of different polarity extracts from the three abovementioned *Hypericum* species. Specifically, phenolic and flavonoid content were assessed and scavenging/reducing and enzyme inhibition properties were assayed, as well. Additionally, the extracts were tested on isolated mouse cortex specimens exposed to the inflammatory stimulus constituted by *Escherichia coli* lipopolysaccharide (LPS) in order to evaluate anti‐inflammatory and neuromodulatory effects. In silico evaluations, including network pharmacology, molecular docking, and dynamics analyses, were also conducted in order to unravel the mechanisms underlying the biopharmacological properties of the extracts. The results confirm the potential applications of bioactive extracts from these species to work as antidepressant agents.

## 2 Materials and Methods

### 2.1 Plant Collection

In 2023, botanical specimens were collected from the city of Elazığ, Turkey. (*H. lysimachioides*: Alibeyköy location; *H. scabrum* : Harput, Gümüşbağlar village; *H. uniglandulosum*: Alibeyköy location). Dr. Ugur Cakilcioglu conducted the taxonomic identification, and a voucher specimen was preserved in the herbarium at Munzur University (voucher numbers: 17‐21 UC, 16‐21 UC, and 22‐11 UC, respectively). The aerial parts were segregated, dried in the shade at ambient temperature, pulverized, and thereafter stored away from light.

### 2.2 Plant Extract Preparation

The extraction procedure included two solvents: a 70% ethanol/water mixture and water. Each 10 g sample was macerated with 200 mL of ethanol/water and water for 24 h at ambient temperature. The aqueous extract was prepared by infusing 10 g of plant material in boiling water for 15 min. Organic solvents were removed via evaporation under low pressure, and the aqueous extract was subjected to freeze‐drying.

### 2.3 Assay for Total Phenolic and Flavonoid Contents

Total phenolics and flavonoids were quantified according to the procedures outlined by (Slinkard and Singleton 1977). Gallic acid (GA) and rutin used as reference standards in the studies, with results expressed as gallic acid equivalents (GAEs) and rutin equivalents (RE).

### 2.4 UHPLC–HRMS Profiling

#### 2.4.1 Chemicals

Acetonitrile (for LC–MS), formic acid (for LC–MS), and methanol (analytical grade) were purchased from Chromasolv (Sofia, Bulgaria). The reference standards used for the compounds' identification were obtained from Extrasynthese (Genay, France) (for gallic, protocatechuic; 4‐hydroxybenzoic; caffeic; *o*‐, *m*‐, and *p*‐coumaric; and vanillic acids; for rutin, myricetin, myricitrin, hyperoside, isoquercitrin, luteolin 7‐*O*‐rutinoside, luteolin 7‐*O*‐glucoside, quercitrin, quercetin, apigenin, kaempferol, orientin, homoorientin, catechin, isovitexin, vitexin) and Phytolab (Vestenbergsgreuth, Bavaria, Germany) (neochlorogenic, chlorogenic, and rosmarinic acids, and hispidulin).

The UHPLC–HRMS analyses of the studied *Hypericum* species were performed as previously described (Gevrenova et al. 2020) on a Q Exactive Plus mass spectrometer (ThermoFisher Scientific Inc.) equipped with a heated electrospray ionization (HESI‐II) probe (ThermoScientific). The equipment was operated in negative ion modes within the *m/z* range from 150 to 1500. The chromatographic separation was achieved on a reversed phase column Kromasil EternityXT C18 (1.8 μm, 2.1 × 100 mm) at 40°C. The mobile phase contained 0.1% formic acid in water (A) and 0.1% formic acid in acetonitrile (B). The run time was 33 min; the flow rate was 0.3 mL/min. The gradient elution program was as follows: 0–1 min, 0%–5% B; 1–20 min, 5%–30% B; 20–25 min, 30%–50% B; 25–30 min, 50%–70% B; 30–33 min, 70%–95%; and 33–34 min, 95%–5%B. Then, the equilibration was 4 min. The injection volume and the flow rate were 1 μL and 300 μL/min, respectively. Data were processed by Xcalibur 4.2 (ThermoScientific, Waltham, MA, USA) instrument control/data handling software and MZmine 2 software.

#### 2.4.2 Assays for In Vitro Antioxidant Capacity

In accordance with the methodologies detailed in our prior publication (Grochowski et al. 2017), various antioxidant tests were carried out. The outcomes were represented as milligrams of Trolox equivalents (TEs) per gram for the DPPH, ABTS radical scavenging, CUPRAC, and FRAP tests. In millimoles of TEs per gram of extract, the phosphomolybdenum (PBD) test examined antioxidant potential, and in milligrams of disodium edetate equivalents (EDTAEs) per gram of extract, the metal chelating activity (MCA) was determined.

#### 2.4.3 Inhibitory Effects Against Some Key Enzymes

In accordance with the established protocols (Grochowski et al. 2017), experiments on enzyme inhibition were performed on the samples. Acarbose equivalents (ACAEs) per gram of extract were used to measure the activities that inhibit amylase and glucosidase, while milligrams of galanthamine equivalents (GALAEs) per gram of extract were used to examine the inhibition of acetylcholinesterase (AChE) and butyrylcholinesterase (BChE). The amount of tyrosinase inhibition for each gram of extract was measured in milligrams of Kojic acid equivalents (KAEs).

#### 2.4.4 Ex Vivo Study

Adult C57/BL6 male mice (3‐month‐old, weight 20–25 g) were housed in Plexiglass cages (two to four animals per cage; 55 × 33 × 19 cm) and maintained under standard laboratory conditions (21°C ± 2°C; 55% ± 5% humidity) on a 14/10 h light/dark cycle, with ad libitum access to water and food. Housing conditions and experimentation procedures were strictly in agreement with the European Community ethical regulations (EU Directive no. 26/2014) on the care of animals for scientific research. In agreement with the recognized principles of “replacement, refinement and reduction in animals in research,” colon specimens were obtained as residual material from vehicle‐treated mice randomized in our previous experiments, approved by the local ethical committee (‘G. d'Annunzio’ University, Chieti, Italy) and Italian Health Ministry (Project No. 885/2018‐PR). Isolated cortex specimens were maintained in a humidified incubator with 5% CO_2_ at 37°C for 4 h (incubation period) in RPMI buffer added with bacterial LPS (50 μg/mL). During the incubation period, the tissues were challenged with scalar concentrations of the extract (50–100 μg/mL).

Finally, as regards COX‐2, NOS‐2, SERT, and NET gene expression, cortex specimens were dissected and stored in RNAlater solution (Ambion, Austin, TX) at −20°C until further processed. Total RNA was extracted from the cortex using TRI Reagent (Sigma‐Aldrich, St. Louis, MO). In all, 1 μg of total RNA extracted from each sample in a 20 μL reaction volume was reverse transcribed using the High Capacity cDNA Reverse Transcription Kit (Applied Biosystems, Foster City, CA, USA). Reactions were incubated in a 2720 Thermal Cycler (Applied Biosystems, Foster City, CA, USA) initially at 25°C for 10 min, then at 37°C for 120 min, and finally at 85°C for 5 s. Gene expression was determined by quantitative real‐time PCR using TaqMan probe‐based chemistry (Applied Biosystems, Foster City, CA, USA). The real‐time PCR was carried out in triplicate for each cDNA sample in relation to each of the investigated genes. Data were elaborated with the Sequence Detection System (SDS) software, Version 2.3 (Applied Biosystems, Foster City, CA, USA).

### 2.5 Screening of Potential Targets

The identification of therapeutic targets represents a pivotal stage in the development of novel pharmaceutical agents within the field of medical research. GeneCards and the Comparative Toxicogenomics Database (CTD) represent invaluable resources for the identification of potential therapeutic targets, particularly in the context of depression, anxiety disorders, and neuroinflammation. By querying these databases with terms related to the selected diseases, a number of associated genes were identified. Furthermore, the PubChem and CTD databases were utilized to conduct a more detailed investigation of the genes associated with these conditions and their respective compounds. To identify common targets across these diseases, the Venny V2.1 web tool (https://bioinfogp.cnb.csic.es/tools/venny/) was employed, which revealed shared critical targets that could play a pivotal role in the therapeutic effects on depression, anxiety disorders, and neuroinflammation (Yagi et al. 2024; Zengin et al. 2024).

### 2.6 KEGG Enrichment Analysis

To comprehensively explore the biological processes and pathways potentially affected by the key targets of *Hypericum* species, a KEGG pathway enrichment analysis was performed. The analysis specifically focused on pathways associated with depression, anxiety disorders, and neuroinflammation, revealing a total of 15 enriched pathways. For this purpose, the Database for Annotation, Visualization, and Integrated Discovery (DAVID, Version 6.8, https://david.ncifcrf.gov/home.jsp) was employed (Huang et al. 2009). The analysis utilized official gene symbols for target identification, with the gene list defined as the input and *Homo sapiens* as the species of interest. Pathways with a *p*‐value of less than 0.05 were considered statistically significant. The enriched pathways were visualized using R software in conjunction with the Pathview plugin (https://pathview.uncc.edu/) in R (V4.3.3) (Yagi et al. 2024; Zengin et al. 2024).

### 2.7 Molecular Docking

In this study, proteins and enzymes were sourced from the Protein Data Bank (PDB). For detailed descriptions of the standard enzymes and relevant proteins, please refer to Table S1, which lists the following sources: AChE, BChE, tyr, amylase, and glucosidase (Yagi et al. 2024; Cetiz et al. 2024; Cusumano et al. 2024; Duran et al. 2024; Kurt‐Celep et al. 2025). The proteins selected for this study were categorized according to their associations with neuromodulation and neuroinflammation. The neuromodulation proteins COX2, NET, NOS2, and SERT were selected based on gene regulation criteria, whereas the proteins associated with neuroinflammation (IL1B, IL6, and PTGS2) were identified through STRING and KEGG pathway analyses. The selection of these proteins was informed by prior research identifying potential therapeutic targets, as well as the results obtained from pathway and KEGG analyses. Upon retrieval, the co‐crystallized ligands, cofactors, and water molecules were removed from the protein structures using BIOVIA Discovery Studio Visualizer V4.5, in order to prepare them for molecular docking studies. The ligands were optimized using OpenBabel V3.1.1 following their download from the PubChem database. Subsequently, the protein and enzyme structures were prepared for molecular docking using MGL Tools V1.5.6. Active binding sites were identified through CavitOmiX V1.0 in PyMOL, POCASA V1.1, or based on inhibitor binding sites documented in the literature (Table S1) (Yu et al. 2010). To validate the accuracy of the docking process, the proteins were re‐docked with their respective ligands and the root‐mean‐square deviation (RMSD) values were calculated. The following formula is used to calculate the RMSD:

$$\text{RMSD}=\sqrt{\frac{1}{N}\sum_{i=1}^{N}{\left(r_{i}^{\mathrm{ref}}-r_{i}^{\text{target}}\right)}^{2}}$$

Molecular docking was conducted using AutoDock Vina V1.1.2, with grid boxes delineated in accordance with the methodology proposed by Trott and Olson (Trott and Olson 2010). The protein–ligand interactions were subjected to further analysis using PLIP, with a particular focus on hydrogen bond interactions in order to ensure the robustness and accuracy of the docking results.

### 2.8 Calculation of MM/PBSA Free Energy to Determine Ligand‐Binding Affinity

The stability and free energy calculations of the molecules were performed using the gmx_MMPBSA tool (https://valdes‐tresanco‐ms.github.io/gmx_MMPBSA/dev/getting‐started/). Initially, molecular dynamics (MD) simulations were conducted for a duration of 10 ns in order to evaluate the stability of each molecule. The compounds exhibiting the highest stability were selected for further analysis. Subsequently, the selected molecules were subjected to 100‐ns MD simulations to gain deeper insights into their behavior and stability over a longer timescale (Miller III et al. 2012; Valdés‐Tresanco et al. 2021).

### 2.9 MD Simulation Setup for Ligand Stability and Flexibility

MD simulations were conducted utilizing the CHARMM graphical user interface (GUI) (https://charmm‐gui.org). The Solution Builder tool was employed for system configuration in accordance with the protocol outlined by Jo et al. (Jo et al. 2008). The CHARMM36m force field was employed for protein parameterization, in accordance with the methodologies outlined by Yagi et al. [28] and Maier et al. (Maier et al. 2015). A periodic boundary condition with TIP3P water molecules was employed to ensure that the minimum distance between the protein and the box edges was 10 Å. In order to neutralize the system and set the NaCl concentration to 0.15 M, counterions were introduced. The Verlet cutoff scheme was employed for the management of electrostatic and van der Waals interactions, while the LINCS algorithm was utilized for the constraint of bond lengths. The long‐range electrostatics were computed using the particle mesh Ewald (PME) method. The minimization of energy was achieved via the steepest descent algorithm until the potential energy was reduced to a value of less than 1000 kJ/mol/nm. The system was then equilibrated under NVT and NPT conditions at 303.3 K in order to ensure thermodynamic stability. Subsequently, production simulations were conducted for a period of 100 ns (nstep = 50,000,000) utilizing the GROMACS 2023.2 software.

### 2.10 Statistical Analysis

Statistical analysis was performed by GraphPad Prism (Version 5.01) software (GraphPad Software Inc., San Diego, CA, USA). The statistical significance (*p* < 0.05) was evaluated through analysis of variance (ANOVA) followed by Newman–Keuls comparison multiple test.

## 3 Results and Discussion

### 3.1 Total Phenolic and Flavonoid Content

Phenolic compounds and flavonoids are secondary metabolites of plants that are widely studied for their biological properties, including antioxidant, anti‐inflammatory, and antimicrobial activities. The determination of total phenolic content (TPC) and total flavonoid content (TFC) is therefore an important starting point for assessing the biological potential of plant extracts. In this study, the TPC and TFC of aqueous and 70% hydroalcoholic extracts of *H. scabrum* , *H. lysimachioides*, and *H. uniglandulosum* were tested. The values are expressed as GAEs per gram of extract for TPC and as rutin equivalents (REs) per gram of extract for TFC. The results, reported in Table 1, indicate that the extract of *H. lysimachioides* obtained with ethanol/water (70%) presents the highest TPC value (69.21 ± 0.78 mg GAE/g), followed by the aqueous extract (65.35 ± 1.53 mg GAE/g). This suggests that both solvents are effective in extracting phenolic compounds from *H. lysimachioides*, though the ethanolic solvent is slightly superior. Similarly, for *H. scabrum* , the phenolic content is higher with ethanol/water (63.17 ± 1.09 mg GAE/g) compared to water (54.56 ± 0.81 mg GAE/g). The extract of *H. uniglandulosum* showed the lowest TPC values with both ethanol/water (42.00 ± 0.34 mg GAE/g) and water (41.25 ± 0.23 mg GAE/g), indicating a lower concentration of phenolic compounds in this species. Overall, the ethanol/water extract proved to be the most effective solvent for all species, although the differences are not particularly pronounced for some species, such as *H. uniglandulosum*. Regarding the TFC, the aqueous extract of *H. uniglandulosum* had the highest TFC (78.70 ± 0.20 mg RE/g), while the water extract of *H. scabrum* contained the lowest TFC (40.54 ± 0.04 mg RE/g). In *H*. *lysimachioides*, the ethanol/water extract reported a high value (72.63 ± 0.81 mg RE/g), though it was lower than the ethanol/water extract of *H. uniglandulosum*. Studies on the TPC and TFC of the *Hypericum* species analyzed here are limited or scarce. Hakimoğlu et al. (Hakimoğlu et al. 2007) determined the TPC of the 70% ethanolic extract of *H. lysimachioides*, obtaining a value of 307 μg of GAE/mg of extract, which is significantly higher than our results. This discrepancy may be due to differences in experimental conditions or extraction methods. Similarly, the TPC of *H. scabrum* reported by Safapour et al. (Safapour et al. 2023), equal to 2.733 mg GAE/g for the 50% ethanolic aqueous extract, is lower than our values, suggesting a possible influence of the different ethanol concentrations or variability in the extract composition.

**TABLE 1:** Total phenolic and flavonoid contents in the tested extracts.*

Solvents	Species	TPC (mg GAE/g)	TFC (mg RE/g)
Ethanol/Water (70%)	*H. lysimachioides*	69.21 ± 0.78^a^	72.63 ± 0.81^b^
	*H. scabrum*	63.17 ± 1.09^b^	67.51 ± 1.10^c^
	*H. uniglandulosum*	42.00 ± 0.34^d^	78.70 ± 0.20^a^
Water	*H. lysimachioides*	65.35 ± 1.53^b^	49.72 ± 0.15^e^
	*H. scabrum*	54.56 ± 0.81^c^	40.54 ± 0.04^f^
	*H. uniglandulosum*	41.25 ± 0.23^d^	53.01 ± 0.15^d^

*Note:* Different letters indicate significant differences between the tested extracts (*p* < 0.05). Abbreviations: GAE, gallic acid equivalent; RE, rutin equivalent.

* Values are reported as mean ± SD of three parallel measurements.

### 3.2 UHPLC–HRMS Profiling

Based on the retention times, MS and MS/MS accurate masses, fragmentation patterns in MS/MS spectra, relative ion abundance, and comparison with reference standards and literature data, a total of 100 secondary metabolites were tentatively annotated or identified in the studied *Hypericum* extracts (Table 2). Thirty compounds from the group of hydroxybenzoic, hydroxycinnamic, acylquinic acids, and derivatives; 61 flavonoids; four xanthones; two benzophenones; one chromone; and two naphtodianthrones (hypericin and pseudo hypericin) were found in the extracts.

**TABLE 2:** UHPLC–HRMS profiling of the studied *Hypericum* species.

No	Identified/tentatively annotated compound	Molecular formula	Exact mass [M‐H]^−^	Fragmentation pattern in (−) ESI‐MS/MS	t_R_ (min)	Δ ppm	Level of confidence	Distribution
**Hydroxybenzoic, hydroxycinnamic, acylquinic acids, and derivatives**								
**1**	Quinic acid *O*‐hexoside	C_19_H_34_O_17_	533.1723	533.1746 (2.6), 191.0553 (100), 173.0444 (0.6), 153.0180 (0.1), 127.0386 (2.0)	0.70	2.29	2	1,2,3,4,5,6
**2**	Citric/isocitric acid	C_6_H_8_O_7_	191.0197	191.0191 (7.7), 173.0082 (1.5), 154.9978 (0.6), 129.0181 (5.7), 111.0074 (100), 85.0280 (28.5)	0.89	−3.33	2	1,2,3,4,5,6
**3**	Hydroxybenzoic acid‐*O*‐hexoside	C_13_H_16_O_8_	299.0772	299.0770 (0.7), 137.0231 (100), 93.0331 (64.5)	1.27	−0.91	2	1,2,3,4,5,6
**4**	Gallic acid *O*‐hexoside	C_13_H_16_O_10_	331.0671	331.0677 (7.9), 169.0133 (100), 125.0231 (42.9), 97.0280 (1.4), 79.0176 (0.3)	1.57	1.78	2	1,2,3,4,5,6
**5**	Protocatechuic acid‐*O*‐hexoside	C_13_H_16_O_9_	315.0722	315.0727 (100), 225.0411 (0.4), 153.0184 (27.7), 152.0103 (55.5), 108.0202 (86.9), 109.0286 (10.3)	1.68	1.69	2	1,2,3,4,5,6
**6**	Vanillyl‐*O*‐hexoside	C_14_H_18_O_9_	329.0878	329.0883 (2.1), 167.0340 (100), 152.0104 (21.2), 123.0438 (13.8), 108.0202 (37.9)	1.74	1.47	2	1,2,3,4,5,6
**7**	Protocatechuic acid	C_7_H_6_O_4_	153.0193	153.0182 (16.7), 109.0281 (100)	2.02	−3.81	1	1,2,3,4,5,6
**8**	Protocatechuic acid‐*O*‐hexoside isomer	C_13_H_16_O_9_	315.0722	315.0733 (55.7), 153.0546 (36.7), 125.0232 (0.2), 123.0438 (50.7), 109.0281 (44.6)	2.08	3.54	2	1,2,3,4,5
**9**	Syringic acid 4‐*O*‐hexoside	C_15_H_20_O_10_	359.0984	359.0991 (8.3), 197.0449 (100), 182.0212 (18.7), 153.0547 (12.3), 138.0311 (25.3), 123.0075 (30.9)	2.28	1.92	2	2,4,5,6
**10**	Neochlorogenic (3‐caffeoylquinic) acid	C_16_H_18_O_9_	353.0878	353.0885 (41.9), 191.0555 (100), 179.0342 (63.1), 161.0233 (3.7), 135.0439 (47.2), 102.0279 (0.6)	2.36	1.97	1	1,2,3,4,5,6
**11**	4‐Hydroxybenzoic acid	C_7_H_6_O_3_	137.0244	137.0232 (100), 119.0124 (2.4), 108.0202 (9.7), 93.0330 (6.9)	2.83	−8.96	1	2,3,4,5,6
**12**	*p*‐Coumaric acid	C_9_H_8_O_3_	163.0401	163.0391 (17.8), 135.0438 (3.0), 119.0488 (100)	3.00	−6.06	1	1,2,3,4,5,6
**13**	5‐*p*‐Coumaroylquinic acid	C_16_H_18_O_8_	337.0929	337.0941 (7.7), 191.0555 (10.2), 173.0447 (3.9), 163.0391 (100), 135.0437 (1.1), 119.0489 (25.9), 111.0436 (1.1), 93.0330 (2.7)	3.01	1.23	2	1,2,3,4,5,6
**14**	Quinic acid	C_7_H_12_O_6_	191.0561	191.0553 (100), 173.0446 (1.8), 127.0388 (3.8), 111.0438 (1.8), 93.0331 (6.6), 85.028 (18.9)	3.18	−4.40	2	1,2,3,4,5,6
**15**	Chlorogenic (5‐caffeoylquinic) acid	C_16_H_18_O_9_	353.0878	353.0888 (4.8), 191.0554 (100), 179.0343 (1.1), 161.0235 (1.5)	3.20	2.85	1	1,2,3,4,5,6
**16**	*m*‐Coumaric acid	C_9_H_8_O_3_	163.0401	163.0390 (7.1), 135.0436 (0.2), 119.0488 (100)	3.35	−6.24	1	1,2,3,4,5,6
**17**	4‐Caffeoylquinic) acid	C_16_H_18_O_9_	353.0878	353.0885 (34.9), 191.0554 (69.2), 179.0341 (63.9), 173.0446 (100), 135.0439 (54.3), 111.0438 (3.8), 93.0331 (20.5)	3.36	1.88	2	1,2,3,4,5,6
**18**	3‐Feruloylquinic acid	C_17_H_20_O_9_	367.1035	367.1041 (20.3), 193.0499 (100), 173.0444 (4.6), 134.0360 (51.9), 127.0332 (5.8), 93.0331 (1.8)	3.42	0.61	2	1,2,3,4,5,6
**19**	Caffeic acid	C_9_H_8_O_4_	179.0350	179.0342 (20.3), 135.0439 (100), 117.0335 (0.7), 107.0124 (4.3)	3.52	−0.80	1	1,2,3,4,5,6
**20**	Ferulic acid 4‐*O*‐hexoside	C_16_H_20_O_9_	355.1035	355.1039 (6.2), 235.0610 (0.9), 193.0499 (100), 178.0263 (15.5), 175.0392 (19.8), 149.0598 (10.7), 134.0361 (35.6)	3.75	1.19	2	1,2,3,4,5,6
**21**	1‐Caffeoylquinic acid	C_16_H_18_O_9_	353.0878	353.0887 (6.4), 191.0554 (100), 179.0346 (0.7), 161.0237 (2.2), 111.0436 (1.7)	3.88	2.39	2	1,2,3,4,5,6
**22**	Shikimic acid	C_7_H_10_O_5_	173.0455	173.0445 (100), 155.0341 (1.8), 127.0392 (1.3), 111.0437 (9.7), 93.0331 (70.2)	4.01	−5.88	2	1,2,3,4,5,6
**23**	4‐*p*‐Coumaroylquinic acid	C_16_H_18_O_8_	337.0929	337.0937 (9.1), 191.0547 (0.4), 173.0445 (100), 163.0390 (19.1), 137.0231 (3.7), 119.0489 (7.7), 109.0279 (0.8), 111.0438 (3.0), 93.0331 (20.0)	4.02	2.28	2	1,2,3,4,5,6
**24**	Vanillic acid	C_8_H_8_O_4_	167.0350	167.0340 (60.8), 152.0103 (100), 123.0438 (5.8), 108.0202 (42.2)	4.12	−6.06	1	1,2,3,4,5,6
**25**	4‐Feruloylquinic acid	C_17_H_20_O_9_	367.1035	367.1031 (13.8), 193.0499 (17.4), 173.0445 (100), 134.0361 (13.6), 127.0386 (0.7), 93.0331 (21.9)	4.37	−0.39	2	1,2,3,4,5,6
**26**	*o*‐Coumaric acid	C_9_H_8_O_3_	163.0401	163.0391 (8.4), 135.0444 (0.3), 119.0489 (100)	4.53	−5.99	1	1,2,3,4,5,6
**27**	Gallic acid	C_7_H_6_O_5_	169.0142	169.0133 (77.4), 151.0025 (100), 125.0231 (25.8), 107.0125 (18.4), 83.0123 (28.9)	5.50	−5.45	1	1,2,3,4,5,6
**28**	3,5‐Dicaffeoylquinic acid	C_25_H_24_O_12_	515.1195	515.1211 (18.8), 353.0884 (83.0), 335.0776 (0.9), 191.0554 (100), 179.0342 (40.2), 173.0444 (3.5), 161.0232 (5.1), 135.0439 (37.4), 127.0387 (1.8), 111.0432 (11.4)	5.84	3.09	2	1,2,3,4,5,6
**29**	4,5‐Dicaffeoylquinic acid^b^	C_25_H_24_O_12_	515.1195	515.1207 (100), 353.0884 (56.9), 335.0777 (1.8), 203.0345 (2.8), 191.0554 (34.0), 179.0341 (63.2), 173.0446 (96.6), 135.0439 (58.4), 111.0436 (4.9)	6.23	2.27	2	1,2,3,4,5,6
**30**	Rosmarinic acid	C_18_H_16_O_8_	359.0772	359.0783 (13.3), 197.0450 (28.3), 179.0342 (12.3), 161.0233 (100), 133.0280 (19.8)	6.34	2.89	1	1,2,3,4,5,6
**Flavonoids**								
**31**	(Epi)Gallocatechin	C_15_H_14_O_7_	305.0667	305.0673 (94.1), 287.0566 (0.5), 261.0774 (8.7), 243.0669 (1.6), 179.0341 (26.7), 165.0183 (22.5), 137.0232 (27.8), 125.0231 (100), 109.0281 (16.7), 151.0336 (0.7), 203.0342 (4.2)	1.74	1.91	2	1,2,3,4
**32**	Catechin	C_15_H_14_O_6_	289.0718	289.0721 (100), 271.0612 (1.6), 245.0818 (37.9), 203.0707 (14.7), 179.0341 (8.9), 151.0389 (7.5), 123.0438 (19.0), 109.0281 (30.6)	3.11	1.10	2	1,2,3,4
**33**	Procyanidin B2	C_30_H_26_O_12_	577.1351	577.1369 (100), 559.1246 (0.8), 425.0890 (67.0), 407.0782 (55.9), 381.0976 (2.3), 289.0722 (60.9), 245.0816 (13.9), 203.0713 (7.7), 161.0234 (23.8), 151.0389 (10.2), 137.0233 (17.4), 125.0231 (86.7), 109.0282 (15.9)	3.58	3.03	2	1,2,3,4
**34**	Naringenin‐6,8‐di‐*C*‐hexoside	C_27_H_32_O_15_	595.1678	595.1686 (100), 475.1269 (4.2), 457.1152 (2.5), 415.1045 (10.9), 385.0939 (33.7), 355.0832 (36.5), 271.0629 (0.8), 163.0024 (1.2), 151.0025 (1.4), 119.0489 (14.8), 107.0125 (2.8)	3.61	2.92	2	1,2,3,4,5,6
**35**	Epicatechin	C_15_H_14_O_6_	289.0718	289.0721 (100), 271.0623 (0.8), 245.0819 (36.5), 203.0707 (16.9), 179.0341 (11.9), 151.0389 (11.1), 123.0437 (20.7), 125.0230 (21.0), 109.0280 (35.2)	3.89	0.99	2	1,2,3,4,6
**36**	Quercetin *O*‐hexosyl‐deoxyhexoside isomer I	C_27_H_30_O_16_	609.1461	609.1480 (100), 447.0944 (9.4), 301.0358 (39.3), 300.0267 (5.2), 271.0252 (28.3), 255.0302 (4.1), 227.0346 (2.3), 178.9979 (1.3), 151.0025 (5.4), 121.0279 (1.1), 107.0125 (1.6)	3.83	3.12	3	1,2,3,4,5,6
**37**	Vicenin II	C_27_H_30_O_15_	593.1512	593.1528 (100), 503.1212 (5.2), 473.1100 (15.9), 455.0999 (1.4), 413.0889 (1.1), 383.0781 (17.7), 353.0674 (32.4), 325.0712 (2.2), 297.0772 (10.6), 253.0885 (0.7), 203.0356 (0.7), 149.0604 (0.7), 117.0332 (3.7), 107.0495 (0.2)	4.01	2.76	2	1,2,3,4,5,6
**38**	Apigenin‐6,8‐di‐*C*‐hexoside	C_27_H_29_O_15_	593.1512	593.1528 (100), 503.1236 (4.5), 473.1100 (16.2), 413.0887 (2.1), 395.0769 (1.9), 383.0780 (22.3), 353.0674 (34.7), 325.0726 (2.1), 297.0768 (11.5), 161.0236 (1.8), 117.0331 (4.1)	4.04	2.66	2	1,2,3,5
**39**	Quercetin *C*‐hexoside	C_21_H_20_O_12_	463.0882	463.0894 (100), 373.0573 (28.8), 343.0464 (90.4), 327.0518 (1.3), 313.0359 (0.4), 301.0355 (5.1), 300.0279 (7.4), 241.0510 (1.5), 229.0513 (0.9), 163.0028 (2.6), 151.0026 (1.1), 149.0233 (18.9), 121.0282 (2.0), 107.0126 (1.2)	4.11	2.51	3	1,2,3,4,5,6
**40**	Quercetin *O*‐hexuronyl‐hexoside	C_27_H_28_O_18_	639.1203	639.1219 (100), 463.0896 (61.4), 301.0357 (32.3), 300.0281 (23.9), 283.0254 (4.2), 255.0300 (7.9), 227.0347 (2.3), 178.9986 (0.3), 151.0028 (0.5)	4.36	2.51	2	1,2,3,4,5,6
**41**	Quercetin *O*‐hexuronyl‐deoxyhexoside	C_27_H_28_O_17_	623.1254	623.1268 (98.9), 447.0941 (37.4), 301.0357 (100), 300.0280 (8.6), 271.0249 (6.3), 243.0300 (1.9), 178.9981 (1.9), 151.0024 (14.8), 121.0278 (2.7), 107.0123 (4.9)	4.42	2.34	2	1,2,3,4
**42**	Myricetin 3‐*O*‐hexoside	C_21_H_20_O_13_	479.0831	479.0841 (100), 317.0298 (15.7), 316.0229 (91.2), 287.0204 (11.7), 271.0252 (19.1), 178.9978 (3.0), 151.0024 (2.9), 107.0122 (1.1), 124.0150 (1.3)	4.49	2.14	2	1,2,3,4,5,6
**43**	Myricetin 3‐*O*‐hexuronide	C_21_H_18_O_14_	493.0624	493.0636 (77.3), 317.0306 (100), 316.0231 (2.2), 299.0204 (3.6), 271.0245 (4.3), 243.0296 (2.6), 178.9977 (15.2), 151.0024 (27.3), 107.0124 (9.3)	4.50	2.48	2	1,2,3,4
**44**	Homoorientin (luteolin 6‐*C*‐glucoside)	C_21_H_20_O_11_	447.0933	447.0944 (100), 357.0623 (46.9), 327.0516 (60.7), 311.0567 (2.9), 299.0565 (11.6), 298.0480 (7.5), 297.0406 (10.1), 285.0407 (6.8), 133.0283 (13.5), 151.0027 (0.4), 107.0125 (0.4)	4.55	2.54	1	2,3,5,6
**45**	Orientin (luteolin 8‐*C*‐glucoside)	C_21_H_20_O_11_	447.0933	447.0945 (92.4), 357.0623 (30.8), 327.0517 (100), 311.0568 (1.5), 297.0408 (12.8), 299.0565 (9.4), 298.0479 (6.0), 285.0410 (4.9), 133.0283 (17.0), 151.0382 (0.4), 107.0122 (0.3)	4.68	2.67	1	1,2,3,4,5,6
**46**	Myricetin 3‐*O*‐pentoside	C_20_H_18_O_12_	449.0726	449.0709 (100), 317.0285 (14.3), 316.0229 (99.2), 287.0203 (15.1), 271.0253 (26.9), 243.0299 (47.4), 178.9977 (3.0), 151.0026 (2.6), 107.0129 (1.9), 124.0155 (3.8)	4.96	−3.76	2	1,2,3,4,5,6
**47**	Myricetin 3‐*O*‐hydroxybutyrylacetylhexoside	C_27_H_28_O_17_	623.1263	623.1263 (100), 521.0944 (10.4), 479.0842 (16.8), 317.0297 (17.4), 316.0228 (73.7), 299.0193 (3.5), 271.0250 (25.8), 178.9981 (3.6), 151.0027 (5.4), 107.0126 (1.9)	4.97	2.05	3	1,2,3,4,5,6
**48**	Quercetin *O*‐hexosyl‐hexuronide	C_27_H_28_O_18_	639.1238	639.1221 (25.3), 477.0685 (58.9), 301.0357 (100), 300.0467 (1.5), 155.0299 (3.5), 211.0397 (1.8), 178.9977 (6.0), 151.0025 (19.8), 121.0280 (6.5), 107.0123 (7.7)	5.00	2.88	2	1,2,3,4,5
**49**	Quercetin *O*‐hexosyl‐deoxyhexoside isomer II	C_27_H_30_O_16_	609.1461	609.1481 (86.7), 447.0943 (68.5), 301.0357 (100), 300.0280 (64.2), 271.0252 (36.9), 211.0397 (1.9), 178.9980 (4.9), 151.0026 (15.7), 121.0284 (3.4), 107.0126 (5.1)	5.05	3.32	2	1,2,3,4,5,6
**50**	Rutin	C_27_H_30_O_16_	609.1461	609.1478 (100), 301.0356 (39.8), 300.0280 (55.1), 271.0251 (31.7), 255.0300 (12.9), 227.0352 (2.9), 211.0395 (0.5), 178.9982 (2.7), 151.0027 (5.9), 121.0283 (1.6), 107.0125 (1.9)	5.08	2.83	1	1,2,3,4,5,6
**51**	myricitrin	C_21_H_20_O_12_	463.0882	463.0894 (100), 317.0296 (23.2), 316.0229 (90.7), 287.0201 (14.1), 271.0251 (24.3), 243.0299 (3.1), 178.9977 (4.2), 151.0023 (5.2), 107.0120 (1.7)	5.09	2.70	1	1,2,3,4,5,6
**52**	Isovitexin	C_21_H_20_O_10_	431.0984	431.0992 (100), 341.0671 (8.4), 311.0567 (94.8), 283.0614 (27.4), 239.0717 (1.7), 211.0755 (0.7), 121.0279 (2.9), 117.0332 (12.0), 161.0235 (4.1)	5.14	3.08	1	1,2,3,4,5,6
**53**	Isoquercitrin	C_21_H_20_O_12_	463.0882	463.0894 (100), 301.0352 (35.4), 300.0280 (79.8), 271.0252 (37.8), 255.0299 (16.0), 243.0299 (9.3), 227.0335 (2.3), 211.0396 (1.2), 178.9976 (2.3), 163.0026 (1.5), 151.0025 (7.4), 121.0280 (0.6), 107.0125 (2.5)	5.19	2.57	1	1,2,3,4,5,6
**54**	Luteolin 7‐*O*‐rutinoside	C_27_H_30_O_15_	593.1512	593.1528 (93.3), 285.0406 (100), 151.0027 (4.2), 133.0282 (4.9), 107.0123 (2.0)	5.22	2.76	1	2,5,6
**55**	Quercetin *O*‐hexuronide	C_21_H_18_O_13_	477.0675	477.0687 (75.8), 301.0358 (100), 300.0287 (0.6), 283.0251 (1.8), 255.0302 (3.6), 211.0398 (2.1), 178.9978 (9.0), 151.0026 (22.2), 107.0125 (7.9), 121.0282 (6.0), 163.0027 (3.7)	5.25	2.51	2	2,5
**56**	Hyperoside	C_21_H_20_O_12_	463.0887	463.0894 (100), 301.0356 (29.7), 300.0286 (53.2), 271.0251 (28.7), 255.0300 (12.6), 243.0301 (7.3), 227.0339 (2.0), 211.0397 (1.1), 178.9979 (2.0), 151.0026 (4.7), 163.0029 (1.2), 121.0278 (0.6), 107.0124 (2.3)	5.29	2.64	1	1,2,3,4,5,6
**57**	Acetylorientin	C_23_H_22_O_12_	489.1038	489.1049 (100), 447.0917 (0.4), 429.0830 (5.9), 339.0515 (12.5), 327.0515 (68.9), 311.0565 (2.1), 297.0408 (12.4), 285.0406 (5.1), 133.0282 (19.1), 151.0025 (0.5), 107.0121 (0.9)	5.30	2.13	2	1,2,3,4
**58**	Quercetin 3‐*O*‐acetylhexoside	C_23_H_22_O_13_	505.0988	505.1000 (100), 463.0891 (1.4), 301.0352 (33.9), 300.0278 (81.5), 343.0459 (0.3), 271.0251 (40.5), 178.9978 (2.9), 163.0027 (1.9), 151.0026 (5.5), 121.0283 (1.0), 107.0125 (2.2)	5.58	2.41	2	1,2,3,4,5,6
**59**	Acetylhomorientin	C_23_H_22_O_12_	489.1038	489.1051 (100), 447.0918 (1.3), 429.0837 (9.3), 339.0515 (14.5), 327.0516 (17.1), 309.0410 (37.4), 311.0576 (2.8), 297.0409 (10.1), 285.0403 (3.2), 133.0282 (21.6)	5.60	2.49	2	1,2,3,4
**60**	Quercetin 3‐*O*‐hydroxybutyrylacetylhexoside	C_27_H_28_O_16_	607.1340	607.1323 (100), 505.1007 (12.7), 463.0894 (26.1), 301.0355 (47.9), 300.0280 (71.9), 271.0252 (38.9), 255.0299 (17.9), 227.0352 (2.1), 178.9979 (2.8), 151.0029 (7.8), 121.0280 (1.2), 107.0123 (2.9)	5.63	2.97	3	1,2,3,4,5,6
**61**	Luteolin 7‐*O*‐glucoside	C_21_H_20_O_11_	447.0933	447.0943 (100), 285.0397 (16.3), 284.0331 (56.3), 255.0300 (34.9), 227.0347 (32.7), 211.0401 (1.5), 151.0026 (2.3), 107.0122 (0.3)	5.64	2.27	1	1,2,3,4,5,6
**62**	Quercetin *O*‐hexosyl‐deoxyhexoside isomer III	C_27_H_30_O_16_	609.1461	609.1479 (100), 301.0358 (74.8), 271.0251 (19.2), 211.0399 (1.4), 178.9978 (5.5), 151.0027 (12.8), 107.0125 (5.4)	5.71	3.02	2	1,2,3,4,5,6
**63**	Quercetin 3‐*O*‐pentoside	C_20_H_18_O_11_	433.0776	433.0784 (100), 301.0355 (73.2), 300.0279 (7.9), 271.0251 (35.7), 255.0301 (16.6), 211.0393 (1.5), 178.9975 (2.6), 151.0025 (8.5), 121.0278 (2.1), 107.0123 (3.1)	5.75	1.84	2	1,2,3,4,5,6
**64**	Acetylisovitexin	C_23_H_22_O_11_	473.1089	473.1099 (100), 413.0887 (15.5), 353.0681 (0.8), 341.0671 (13.2), 377.0675 (0.4), 323.0566 (4.9), 311.0566 (8.1), 293.0457 (1.8), 283.0615 (24.9), 269.0459 (4.1), 239.0711 (1.4), 211.0759 (0.9), 151.0031 (0.2), 117.0332 (14.3), 107.0120 (0.6)	5.78	1.96	2	1,2,3,4
**65**	Myricetin 3‐*O*‐acetyldeoxyhexoside	C_23_H_22_O_13_	505.0988	505.1003 (100), 463.0876 (0.3), 445.0792 (1.2), 317.0292 (13.7), 316.0229 (73.3), 301.0346 (6.5), 300.0281 (26.2), 287.0202 (11.7), 271.0252 (29.7), 255.0299 (5.0), 243.0300 (5.7), 178.9976 (3.7), 151.0025 (4.9), 107.0124 (1.6)	5.82	3.12	2	1,2,3,4,5,6
**66**	Kaempferol 3‐*O*‐hexuronide	C_23_H_22_O_13_	461.0725	461.0736 (48.8), 285.0407 (100), 229.0502 (7.2), 211.0401 (1.6), 151.0021 (0.5), 135.0070 (1.3), 107.0125 (2.3)	5.84	2.17	2	1,2,3,4
**67**	Quercitrin	C_21_H_20_O_11_	447.0933	447.0944 (100), 301.0358 (52.8), 300.0280 (60.3), 271.0252 (23.3), 243.0299 (6.4), 227.0350 (1.9), 211.0402 (1.1), 178.9977 (3.1), 151.0025 (7.5), 121.0281 (1.8), 107.0125 (2.9)	5.90	2.54	1	1,2,3,4,5,6
**68**	Isorhamnetin 3‐*O*‐glucoside	C_22_H_22_O_12_	477.1038	477.1049 (100), 315.0502 (8.9), 314.0440 (48.4), 299.0203 (3.1), 271.0251 (19.7), 257.0458 (4.2), 243.0299 (21.9), 227.0348 (3.3), 215.0348 (3.2), 199.0394 (3.7), 151.0025 (2.6), 107.0120 (0.9)	6.02	2.31	2	1,2,3,5,6
**69**	Kaemferol 3‐*O*‐pentoside	C_20_H_18_O_10_	417.0827	417.0836 (100), 285.0396 (15.9), 284.0330 (66.6), 255.0300 (41.2), 227.0348 (39.2), 211.0397 (1.2), 151.0026 (1.8), 135.0069 (0.6), 107.0118 (0.6)	6.07	1.99	2	1,2,3,4,5,6
**70**	Myricetin 3‐*O*‐acetylhexuronide	C_23_H_20_O_15_	535.0729	535.0730 (100), 475.0484 (3.6), 317.0305 (56.9), 316.0224 (28.2), 299.0204 (9.9), 271.0257 (11.1), 243.0298 (3.5), 178.9976 (12.7), 151.0025 (17.2), 107.0124 (6.3)	6.09	0.11	2	1,2,3,4
**71**	Kaempferide 3‐*O*‐dihexoside	C_27_H_30_O_15_	593.1512	593.1528 (67.3), 299.0563 (100), 284.0329 (76.1), 271.0258 (0.2), 227.0352 (1.3), 211.0394 (0.5), 151.0029 (1.7), 107.0126 (0.9)	6.11	2.76	2	1,2,3,4,5,6
**72**	Acetylvitexin	C_23_H_22_O_11_	473.1089	473.1099 (42.7), 413.0887 (72.9), 353.0681 (2.4), 323.0562 (5.3), 311.0567 (4.3), 293.0460 (100), 283.0611 (1.5), 269.0463 (2.2), 149.0226 (0.7), 117.0332 (25.5)	6.12	1.96	2	1,2,3,4
**73**	Myricetin	C_15_H_10_O_8_	317.0303	317.0305 (100), 271.0252 (1.4), 227.0345 (0.7), 178.9978 (27.4), 151.0026 (30.6), 107.0124 (11.2)	6.25	0.82	1	1,2,3,4,5,6
**74**	Luteolin 7‐*O*‐acetylhexoside	C_23_H_22_O_12_	489.1038	489.1051 (100), 327.0518 (3.7), 285.0399 (24.1), 284.0330 (77.4), 255.0300 (38.4), 227.0348 (26.1), 211.0399 (1.6), 151.0221 (1.9), 133.0278 (1.3), 107.0124 (0.8)	6.28	2.35	2	1,2,3,4,5,6
**75**	Kaempferol 3‐*O*‐acetylhexoside	C_23_H_22_O_12_	489.1038	489.1051 (100), 327.0515 (0.4), 285.0406 (67.8), 284.0330 (69.9), 255.0300 (46.3), 227.0347 (28.6), 211.0395 (1.9), 151.0023 (1.6), 135.0074 (1.32), 107.0124 (1.8)	6.29	2.32	2	1,2,3,4,5,6
**76**	Kaempferol 3‐*O*‐hydroxybutyrylacetylhexoside	C_27_H_28_O_15_	591.1391	591.1379 (100), 529.1369 (5.8), 489.1057 (16.0), 447.0947 (19.4), 327.0505 (0.7), 285.0406 (81.9), 284.0330 (53.9), 255.0300 (40.1), 227.0348 (19.4), 151.0027 (1.9), 135.0077 (0.9), 107.0123 (0.1)	6.30	4.06	3	1,2,4,5,6
**77**	Quercetin 4'‐*O*‐hexoside	C_21_H_20_O_12_	463.0882	463.0893 (92.3), 301.0358 (100), 300.0283 (6.8), 271.0247 (4.2), 255.1601 (1.5), 227.0349 (0.9), 178.9978 (14.9), 163.0026 (0.5), 151.0026 (38.2), 121.0282 (8.9), 107.0124 (14.2)	6.48	2.42	2	1,2,3,4,5,6
**78**	Kaempferol 3‐*O*‐deoxyhexoside	C_21_H_20_O_10_	431.0984	431.0991 (100), 285.0406 (75.7), 284.0331 (52.6), 255.0300 (35.6), 227.0347 (32.9), 211.0399 (2.2), 135.0072 (1.2), 107.0119 (1.4)	6.58	3.01	2	1,2,3,4,5,6
**79**	Quercetin 3‐*O*‐malonyldeoxyhexoside	C_24_H_22_O_14_	533.0937	533.0891 (2.8), 489.1051 (100), 447.0945 (1.6), 386.0679 (0.4), 301.0355 (39.5), 300.0281 (68.5), 271.0253 (29.5), 255.0301 (14.4), 227.0347 (2.5), 178.9973 (2.9), 151.0028 (6.4), 121.0279 (1.2), 107.0126 (2.2)	6.68	−4.57	2	1,2,3,4,5,6
**80**	Quercetin 3‐*O*‐acetylhexuronide	C_23_H_20_O_14_	519.0780	519.0791 (100), 459.0583 (5.1), 301.0357 (71.4), 300.0278 (16.8), 283.0257 (1.9), 271.0251 (8.6), 227.0345 (2.1), 211.0398 (1.7), 178.9977 (6.5), 151.0025 (16.5), 121.0281 (4.6), 107.0124 (5.8)	6.76	2.06	2	1,2,3,4
**81**	Quercetin 3‐*O*‐acetyldeoxyhexoside	C_23_H_22_O_12_	489.1038	489.1051 (100), 447.0935 (0.5), 301.0355 (44.5), 300.0280 (76.6), 271.0252 (36.7), 255.0301 (18.4), 227.0345 (3.7), 211.0398 (1.3), 178.9980 (3.7), 151.0025 (8.2), 121.0281 (2.0), 107.0124 (3.1)	7.34	2.35	2	1,2,3,4,5,6
**82**	Luteolin 7‐*O*‐acetylhexuronide	C_23_H_20_O_13_	503.0831	503.0844 (76.7), 443.0638 (6.5), 285.0406 (100), 255.0301 (4.8), 229.0505 (10.3), 151.0027 (2.1), 113.0230 (7.8), 107.0125 (2.3)	7.35	2.52	2	1,2,3,4
**83**	Luteolin	C_15_H_10_O_6_	285.0405	285.0407 (100), 241.0499 (0.3), 217.0505 (1.0), 151.0027 (3.7), 133.0282 (21.0), 121.0280 (0.9), 107.0125 (3.6)	7.58	0.87	2	1,2,3,4,5,6
**84**	Quercetin	C_15_H_10_O_7_	301.0354	301.0357 (100), 273.0412 (2.6), 257.0450 (0.9), 229.0505 (0.7), 178.9977 (19.5), 151.0025 (43.1), 121.0281 (12.8), 107.0124 (14.4)	7.59	1.21	1	1,2,3,4,5,6
**85**	Apigenin	C_15_H_10_O_5_	269.0455	269.0457 (100), 225.0550 (1.4), 151.0025 (5.5), 149.0233 (4.9), 117.0332 (17.3), 107.0124 (5.2)	8.60	0.49	1	1,2,3,4,5
**86**	Kaempferol	C_15_H_10_O_6_	285.0405	285.0407 (100), 257.0452 (0.7), 229.0499 (0.9), 211.0392 (1.0), 151.0026 (1.7), 161.0033 (0.5), 135.0076 (0.3), 107.0123 (1.0)	8.83	0.77	1	1,2,3,4,5,6
**87**	Quercetin 3‐*O*‐coumaroyl‐deoxyhexoside	C_30_H_26_O_13_	593.1301	593.1318 (100), 447.0947 (13.7), 301.0354 (45.2), 300.0280 (63.3), 271.0251 (30.2), 255.0300 (14.1), 211.0398 (2.1), 178.9978 (9.0), 151.0026 (22.2), 107.0125 (7.9), 121.0282 (6.0), 163.0027 (3.7)	9.10	2.99	2	2,3,4,5,6
**88**	Biapigenin	C_30_H_18_O_10_	537.0827	537.0841 (100), 493.0936 (1.1), 386.0769 (3.2), 385.0726 (31.5), 267.0305 (2.8), 223.0403 (3.5), 152.0057 (2.2), 151.0025 (66.2), 117.0332 (7.9), 107.0124 (15.9)	9.35	2.59	2	1,2,3,4,5,6
**89**	Amentoflavone	C_30_H_18_O_10_	537.0827	537.0842 (100), 493.0906 (0.4), 376.0546 (4.3), 375.0518 (54.4), 223.0401 (1.9), 151.0025 (3.1), 117.0332 (7.9), 107.0123 (2.5)	9.98	2.70	2	1,2,3,4,5,6
**90**	Hispidulin (scutellarein‐6‐methyl ether)	C_16_H_12_O_6_	299.0563	299.0564 (65.6), 284.0329 (100), 255.0305 (1.0), 227.0344 (2.6), 211.0394 (1.3), 165.9901 (0.6), 136.9868 (14.0), 117.0329 (1.8)	8.84	1.07	1	1,2,3,4,5,6
**91**	Kaempferide	C_16_H_12_O_6_	299.0561	299.0564 (100), 284.0329 (81.1), 271.0253 (8.5), 243.0306 (0.5), 227.0347 (3.4), 211.0406 (0.4), 151.0026 (3.6), 107.0124 (3.1)	8.93	0.97	2	1,2,3,4,5,6
**Xanthones, benzophenones and chromones**								
**92**	Maclurin *O*‐hexoside	C_19_H_20_O_11_	423.0933	423.0942 (92.6), 261.0408 (100), 243.0309 (3.5), 169.0141 (2.9), 151.0025 (68.1), 125.0232 (6.1), 109.0281 (41.5), 107.0125 (25.9)	2.23	2.12	2	1,2,3,4,5,6
**93**	Mangiferin/isomangiferin	C_19_H_18_O_11_	421.0776	421.0786 (100), 403.0685 (1.3), 331.0466 (62.9), 313.0359 (6.4), 301.0358 (88.9), 271.0252 (19.9), 259.0244 (13.9), 215.0339 (2.0), 125.0231 (1.5), 109.0283 (2.0), 161.0231 (1.0)	3.96	2.39	2	1,2,3,4,5,6
**94**	Mangaphenone *O*‐deoxyhexoside	C_20_H_22_O_10_	421.1140	421.1143 (28.5), 275.0565 (100), 257.0453 (2.1), 243.0300 (56.9), 231.0658 (1.9), 199.0395 (24.3), 175.0392 (15.0), 155.0493 (7.3), 131.0489 (4.2), 109.0279 (1.3)	4.38	0.71	2	1,2,3,4,5,6
**95**	Norathyriol *O*‐hexoside	C_19_H_18_O_11_	421.0776	421.0786 (9.1), 259.0248 (100), 215.0346 (6.2), 187.0391 (2.5), 159.0439 (0.8), 115.0534 (0.2)	5.44	2.39	2	1,2,3,4
**96**	5‐hydroxy‐2‐isopropylchromone‐7‐*O*‐hexoside	C_18_H_22_O_9_	381.1191	427.1235 (4.3) (M‐H + HCOOH), 381.1198 (7.9), 261.0771 (0.3), 219.0658 (100), 204.0424 (6.5), 175.0768 (0.2), 145.0287 (0.2)	6.59	−2.61	2	1,2,3,4,5,6
**97**	Trihydroxymethoxyxanthone *O*‐hexoside isomer I	C_20_H_22_O_11_	435.0933	435.0942 (100), 273.0407 (79.1), 258.0171 (44.6), 229.0140 (9.5), 201.0188 (23.5), 245.0098 (0.5), 185.0226 (0.4), 173.0226 (0.4), 145.0285 (0.5)	7.16	2.06	2	1,2,3,4,5,6
**98**	Trihydroxymethoxyxanthone *O*‐hexoside isomer II	C_20_H_22_O_11_	435.0933	435.0941 (46.0), 273.0406 (100), 258.0170 (60.9), 245.0096 (0.2), 229.0145 (1.8), 201.0187 (5.2), 185.0228 (0.3)	7.61	2.13	2	1,2,3,4,5,6
**Naphthodianthrones**								
**99**	Pseudohypericin	C_30_H_16_O_9_	519.0722	519.0733 (100), 503.0424 (0.6), 487.0465 (2.9), 475.0450 (0.4), 449.0562 (0.7), 431.0591 (0.2), 421.0750 (0.6), 403.0588 (0.2)	16.91	2.32	3	1,2,3,4
**100**	Hypericin	C_30_H_16_O_8_	503.0772	503.0785 (100), 487.0456 (0.9), 459.0883 (1.0), 433.0745 (2.0), 405.0784 (2.6), 361.0884 (0.3), 276.2488 (0.2)	20.77	2.48	3	2,3

*Note:* 1‐ *Hypericum lysimachioides—*ethanol–water extract, 2‐ *Hypericum lysimachioides*—water extract; 3‐ *Hypericum scabrum*—ethanol–water extract; 4‐ *Hypericum scabrum—*water extract; 5‐ *Hypericum uniglandulosum*—ethanol–water extract; 6‐ *Hypericum uniglandulosum*—water extract.

Flavonoids are the largest group of compounds found in the tested species. The MS/MS spectrum of 31 with [M‐H]‐ at m/z 305.067 was acquired (Table 2). The prominent fragment ions at *m/z* 261.077 resulted from the CO_2_ loss and the A ring cleavage, and subsequent ethenone (C_2_H_2_O) loss at *m/z* 219.066. The neutral ring‐B loss gave an abundant ion at *m/z* 179.034 (26.7%), while the base peak at *m/z* 125.021 referred to the ring‐A of the flavanolic skeleton after a heterocycle ring cleavage. Accordingly, 31 was annotated as (epi)gallocatechin (Gevrenova et al. 2024).

**FIGURE 1:**
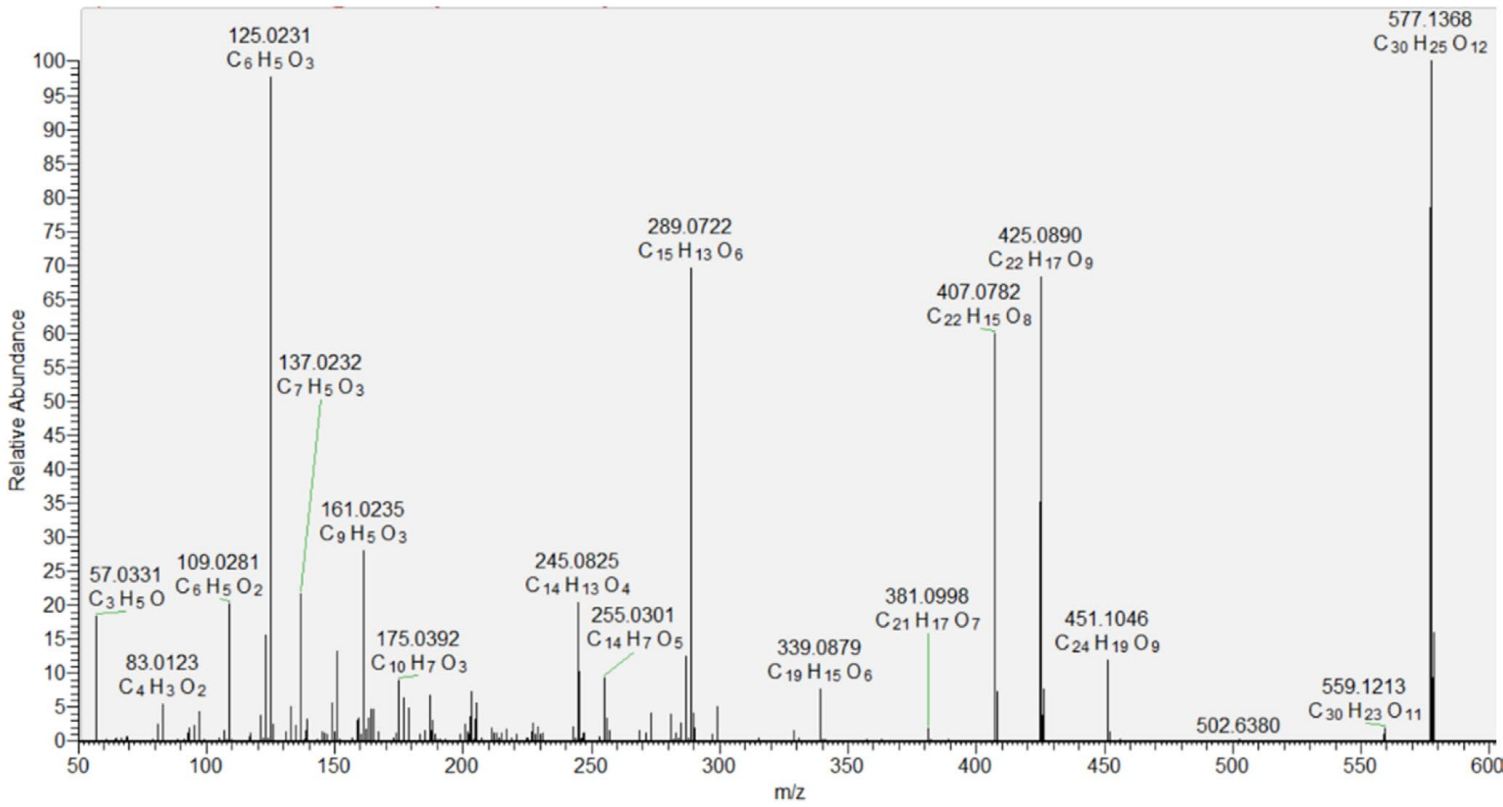
MS/MS spectrum of procyanidin B2 (33).

Compounds 32 and 35 shared the same [М‐Н]^−^ at *m/z* 289.072, consistent with С_15_Н_14_О_7_ and fragment ions at *m/z* 245.082 [М‐Н‐CO_2_]^−^, 179.034 [M‐H‐C_6_H_6_O_2_], 125.023 (^1,4^A^−^), 137.023 (^1,3^A^−^), and 109.0280 [М‐Н‐C_9_H_8_O_4_]^−^ (https://doi.org/10.3390/ph14030266). Compound 32 was identified as catechin based on the comparison of an authentic standard, while 35 was annotated as its isomer epicatechin. A similar fragmentation pathway was presented by 33, the dimer procyanidin B2 (Figure 1) (Marinov et al. 2024).

In the MS/MS spectra of compounds 37, 38, 52, 64, and 72, the aglycone was identified as the flavone apigenin (85) based on the RDA ions ^1,3^B^−^ at *m/z* 117.033, ^1,3^A^−^ at *m/z* 151.003, and ^0.4^A^−^ at *m/z* 107.012. Luteolin (83) and its *O*‐glycosides (54, 61, 74, and 82) and *C*‐glycosides (44, 45, 57, 59) were also found in the studied extracts.

**FIGURE 2:**
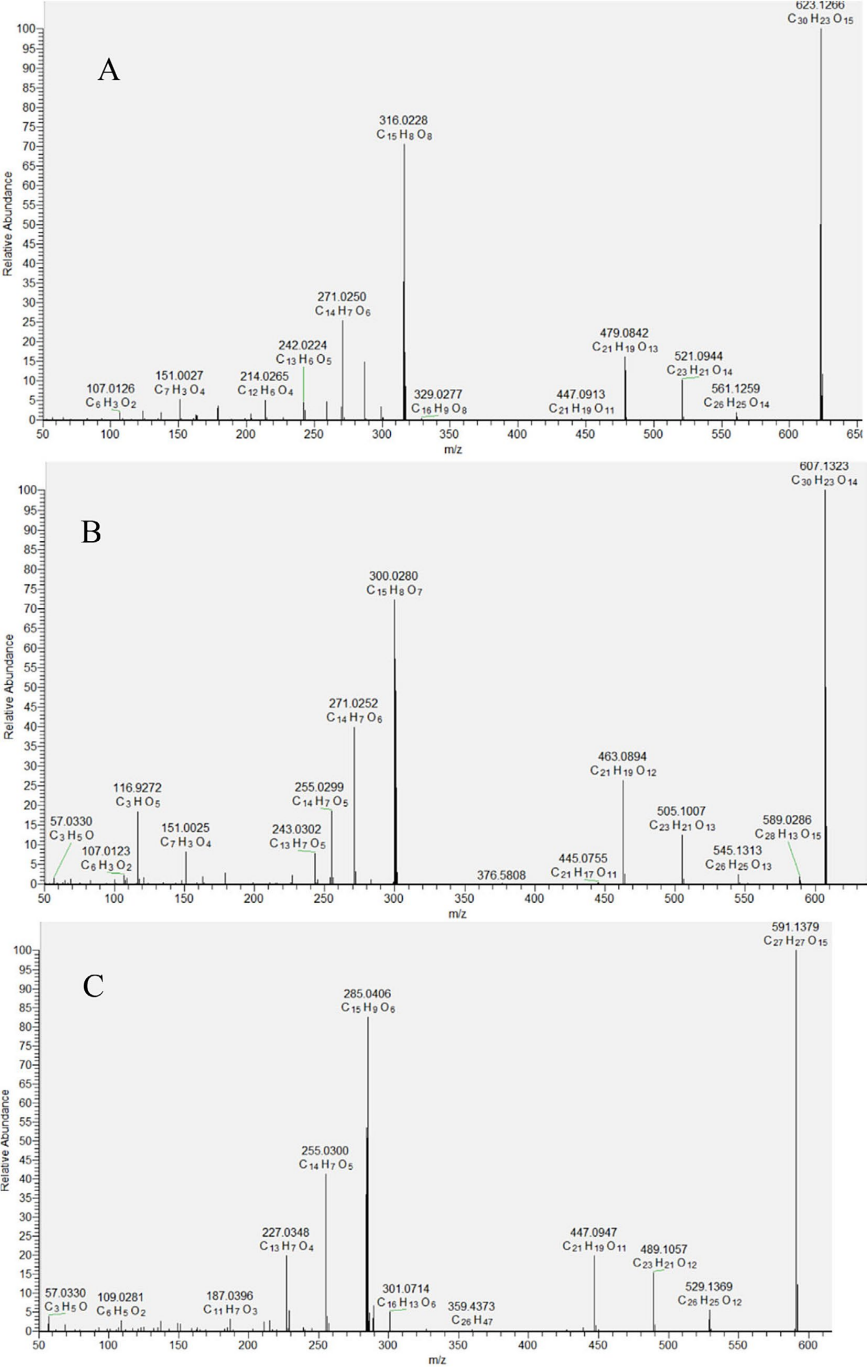
MS/MS spectra of compounds 47 (A), 60 (B), and 76 (C).

The identification of the aglycone luteolin was determined based on a series of fragment ions at *m/z* 285.041 [Lu‐H]^−^, 255.030 [Lu‐H‐CH_2_O]^−^, 257.042 [Lu‐H‐CO]^−^, 241.051 [Lu‐H‐CO_2_]^−^, 227.034 [Lu‐H‐CH_2_O‐CO]^−^, and 211.039 [Lu‐H‐H_2_O‐2CO]^−^, together with RDA ions ^1,3^B^−^ at *m/z* 133.029, ^1,3^A^−^ at *m/z* 151.003, and ^0.4^A^−^ at *m/z* 107.012 (Table 2). Kaempferol (86) and its *O*‐glycosides (69, 71, 75, 76, 78) were dereplicated by fragment ions at *m/z* 285.041 [K‐H]^−^, 135.008 (^0,3^A^−^), 151.003 (^1,3^A^−^), and 107.012 (^0.4^A^−^). Quercetin (84) and its *C*‐ (39) and *O*‐glycosides (40, 41, 48, 49, 50, 53, 55, 56, 58, 60, 62, 63, 67, 77, 79, 80, 81, 87) were annotated based on fragment ions at *m/z* 301.036 [Q‐H]^−^, 257.045 [Q‐H‐CO_2_]^−^, 229.051 [Q‐H‐CO_2_‐CO]^−^, 178.998 (^1,2^A^−^), 121.028 (^1,2^B^−^), 151.003 (^1,3^A^−^), and 107.012 (^0.4^A^−^). Similarly, myricetin (73) and its *O*‐glycosides (42, 43, 46, 47, 51, 65, and 70) were tentatively identified by fragment ions at *m/z* 317.031 [Myr‐H]^−^, 178.998 (^1,2^A^−^), 151.003 (^1,3^A^−^), and 107.012 (^0.4^A^−^). An important step in the dereplication/annotation of flavonoid glycosides was the neutral loss of 162.05, 146.05, 132.04, 176.03, 204.06, 188.07, 218.04, 232.05,338.09, 322.089, 308.11, and 294.09 Da, corresponding to hexose, deoxyhexose, pentose, hexuronic acid, acetylhexose, acetyldeoxyhexose, acetylhexuronic acid, malonylhexose, hexuronylhexose, hexuronyldeoxyhexose, hexosyldeoxyhexose/rutinose, and dihexose, respectively (Zheleva‐Dimitrova et al. 2023). Compounds 47 [M‐H]^−^ at *m/z* 623.126, 60 [M‐H]^−^ at *m/z* 607.134, and 76 [M‐H]^−^ at *m/z* 591.139 revealed a similar fragmentation pathway, including subsequent losses of 102.03, 42.01, and 162.05, corresponding to hydroxybutyryl, acetyl, and hexosyl moiety, respectively. Based on the different aglycon ion, compounds were related to hydroxybutyrylacetylhexosides of myricetin (47), quercetin (60), and kaempferol (76), respectively (Table 2; Figure 2).

**FIGURE 3:**
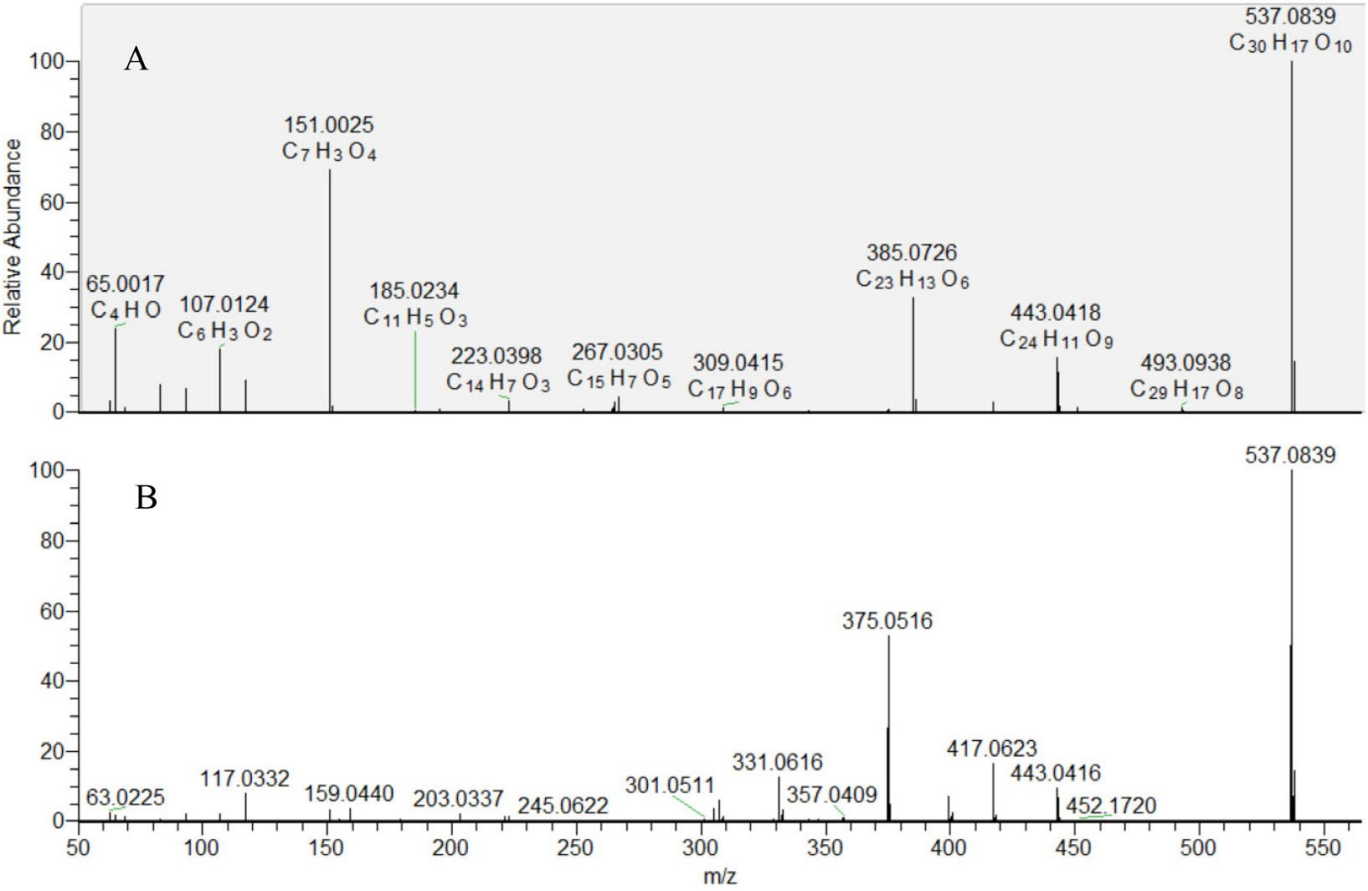
MS/MS spectra of compounds 88 (A) and 89 (B).

Two isobars (88 and 89) shared the same deprotonated molecule [M‐H]^−^ at *m/z* 537.083. Compound 88 revealed a prominent ion at *m/z* 385.07, while 89 gave a fragment at *m/z* 375.05. Based on comparison with previously described fragmentation pathways, compounds 88 and 89 were identified as biapigenin and amentoflavone, respectively [DOI 10.1002/pca.1249] (Figure 3).

Xanthones, benzophenones, and chromones (92–98) were tentatively identified based on comparison to previously found *Hypericum* secondary metabolites from these classes (Marinov et al. 2024; See et al. 2014; Trevisan et al. 2016). Compound 100 [M‐H]^−^ at *m/z* 503.0772 gave fragment ions at *m/z* 487.046 [M‐H‐CH_4_]^−^, 459.089 [M‐H‐CH4‐CO]^−^, 433.074 [M‐H‐CH_4_‐CO‐C_2_H_2_]^−^, and 405.078 [M‐H‐CH_4_‐2CO‐C_2_H_2_]^−^. This fragmentation pathway was previously described, and compound 100 was annotated as hypericin (Riedel et al. 2004). Compound 99 differed from 100 by one OH group and revealed a similar MS/MS spectrum. Therefore, 99 was ascribed to pseudohypericin (Table 2).

### 3.3 Antioxidant Effects

The antioxidant activity of a plant extract can be evaluated using various complementary assays, such as DPPH, ABTS, CUPRAC, FRAP, metal chelation, and PBD, as these methods measure different aspects of antioxidant activity, such as the ability to donate electrons or hydrogen, or the capacity to chelate metals. This multi‐method approach is useful as it provides a comprehensive understanding of the antioxidant potential of plant extracts. In this study, aqueous and 70% hydroalcoholic extracts of three *Hypericum* species were tested for scavenging capacity using DPPH and ABTS assays; reducing capacity using CUPRAC, FRAP, and PBD assays; and metal chelation capacity. The results are reported in Table 3. Regarding quenching activity assays, in the DPPH assay, the ethanol/water extract of *H. lysimachioides* exhibited the highest antioxidant activity (224.80 ± 0.86 mg TE/g), followed by the ethanol/water extract of *H. scabrum* (189.35 ± 4.81 mg TE/g). The aqueous extract of *H. lysimachioides* and *H. scabrum* showed lower values (126.76 ± 6.98 mg TE/g and 95.81 ± 2.97 mg TE/g, respectively) compared to the ethanolic solvent, suggesting better efficiency of ethanol/water in extracting antioxidant compounds. *H. uniglandulosum* had the lowest values with both solvents (102.11 ± 1.77 mg TE/g for ethanol/water and 71.48 ± 1.30 mg TE/g for water), indicating a lower quantity of active antioxidant compounds in this species. In the ABTS assay, the ethanol/water extract of *H. lysimachioides* showed the highest value (313.00 ± 8.86 mg TE/g), followed by the ethanol/water extract of *H. scabrum* with a value of 257.89 ± 28.62 mg TE/g and then the water extract of *H. lysimachioides* (197.43 ± 4.57 mg TE/g). Other extracts showed lower scavenging ability (170.78 ± 3.20 mg TE/g for the *H. scabrum* water extract, 155.91 ± 1.50 mg TE/g for the ethanol/water *H. uniglandulosum* extract, and 100.01 ± 2.50 mg TE/g for the *H. uniglandulosum* water extract). Regarding reducing capacity, in the CUPRAC assay, the best result was obtained by the ethanol/water extract of *H. lysimachioides* (462.93 ± 3.70 mg TE/g), while the aqueous extract reported a lower value (357.81 ± 7.56 mg TE/g). *H. scabrum* showed relatively high values with both ethanol/water (365.63 ± 18.02 mg TE/g) and water (335.53 ± 6.77 mg TE/g). *H. uniglandulosum* exhibited the lowest values, with the ethanol/water extract (225.99 ± 8.34 mg TE/g) being superior to the aqueous extract (164.91 ± 2.46 mg TE/g). In the FRAP assay, *H. lysimachioides* demonstrated the highest reducing capacity with the ethanol/water solvent (273.18 ± 3.83 mg TE/g). Its water extract showed a value of 223.34 ± 2.57 mg TE/g. *H. scabrum* also showed good reducing activity, with ethanol/water (235.06 ± 7.57 mg TE/g) and water (210.56 ± 2.19 mg TE/g). *H. uniglandulosum* had significantly lower values in both cases (128.81 ± 5.81 mg TE/g for ethanol/water and 101.93 ± 0.56 mg TE/g for water). In the PBD assay, *H. uniglandulosum* reported the highest value with ethanol/water (3.13 ± 0.41 mmol TE/g). Its water extract exhibited a lower value (2.03 ± 0.03 mmol TE/g). *H. lysimachioides* also showed good activity with the aqueous solvent (2.74 ± 0.05 mmol TE/g), while *H. scabrum* had slightly lower activity with both solvents (2.29 ± 0.11 mmol TE/g for ethanol/water and 2.26 ± 0.02 mmol TE/g for water). The metal chelation activity was particularly high in the aqueous extract of *H. uniglandulosum* (20.94 ± 0.16 mg EDTAE/g), followed by the aqueous extract of *H. scabrum* (20.82 ± 0.08 mg EDTAE/g). This suggests that the aqueous extracts of these species contain compounds with a strong affinity for metals. On the other hand, the water extract of *H. lysimachioides* showed a lower value (7.50 ± 0.54 mg EDTAE/g). Regarding the ethanol/water extracts, the values were 16.76 ± 0.32 mmol TE/g, 8.36 ± 0.50 mmol TE/g, and 6.66 ± 0.54 mmol TE/g for *H. uniglandulosum*, *H. lysimachioides*, and *H. scabrum* , respectively. The results indicate that both aqueous and hydroalcoholic extracts of *H. lysimachioides* were the most successful in almost all assays, followed by the extracts of *H. scabrum* and finally *H. uniglandulosum*. This could be explained by the higher phenolic content in *H. lysimachioides* extracts and the lower content found in *H. uniglandulosum* extracts, as reported in the Folin–Ciocalteu assay (Table 1). Our experimental results are generally in line with the literature. For example, for *H. lysimachioides*, the high values observed in the DPPH and ABTS assays align with Hakimoğlu et al. (Hakimoğlu et al. 2007), who reported an IC50 of 28 μg/mL for the ethanolic extract in the DPPH assay. In the literature, other studies of different extracts obtained from species belonging to the *Hypericum* genus are present. For *H. scabrum* , Keser et al. (Keser et al. 2020) reported that the ethanol (ABTS: 98.99% and OH: 97.33% at 500 μg/mL) and water extracts (ABTS: 97.89% and OH: 96.36% at 500 μg/mL) from the flowers showed strong scavenging activities against ABTS and OH radicals, with the water extract (91.66% at 500 μg/mL) demonstrating better DPPH radical scavenging activity than the standard antioxidant BHA (90.16% at 500 μg/mL). Additionally, Shafaghat (Shafaghat 2011) found that hexane extracts from different parts of the plant, such as flowers, leaves, stems, and seeds, showed considerable antioxidant activity, with the highest radical scavenging activity detected in the seed extract. In a 2022 study, the methanol extract from the flowers of *H. scabrum* exhibited the highest antioxidant activity compared to solvents like acetone, ethyl acetate, and water (Ergin et al. 2022). Regarding *H. uniglandulosum*, Turkoglu et al. (2015) highlighted that the methanol extract had a strong capacity to neutralize free radicals in the DPPH assay, while the water extract also showed significant antioxidant properties. Both water and methanol extracts demonstrated notable metal chelating ability, contributing to their overall antioxidant potential. Studies conducted on this genus, including ours, suggest that it is worth further exploring the antioxidant capacity of other species in the *Hypericum* genus, which, after further investigation and in‐depth studies, could become candidates for the future development of drugs aimed at combating oxidative stress.

**TABLE 3:** Antioxidant properties of the tested extracts.*

Solvents	Species	DPPH (mg TE/g)	ABTS (mg TE/g)	CUPRAC (mg TE/g)	FRAP (mg TE/g)	Chelating (mg EDTAE/g)	PBD (mmol TE/g)
Ethanol/Water (70%)	*H. lysimachioides*	224.80 ± 0.86^a^	313.00 ± 8.86^a^	462.93 ± 3.70^a^	273.18 ± 3.83^a^	8.36 ± 0.50^c^	2.40 ± 0.19^bc^
	*H. scabrum*	189.35 ± 4.81^b^	257.89 ± 28.62^b^	365.63 ± 18.02^b^	235.06 ± 7.57^b^	6.66 ± 0.54^d^	2.29 ± 0.11^bc^
	*H. uniglandulosum*	102.11 ± 1.77^d^	155.91 ± 1.50^d^	225.99 ± 8.34^d^	128.81 ± 5.81^d^	16.76 ± 0.32^b^	3.13 ± 0.41^a^
Water	*H. lysimachioides*	126.76 ± 6.98^c^	197.43 ± 4.57^c^	357.81 ± 7.56^bc^	223.34 ± 2.57^b^	7.50 ± 0.54^cd^	2.74 ± 0.05^ab^
	*H. scabrum*	95.81 ± 2.97^d^	170.78 ± 3.20^cd^	335.53 ± 6.77^c^	210.56 ± 2.19^c^	20.82 ± 0.08^a^	2.26 ± 0.02^bc^
	*H. uniglandulosum*	71.48 ± 1.30^e^	100.01 ± 2.50^e^	164.91 ± 2.46^e^	101.93 ± 0.56^e^	20.94 ± 0.16^a^	2.03 ± 0.03^c^

*Note:* Different letters indicate significant differences between the tested extracts (*p* < 0.05). Abbreviations: EDTAE, EDTA equivalent; MCA, metal chelating activity; PBD, phosphomolybdenum; TE, trolox equivalent.

* Values are reported as mean ± SD of three parallel measurements.

### 3.4 Enzyme Inhibitory Effects

The inhibition of specific enzymes, such as AChE, BChE, tyrosinase, amylase, and glucosidase, is of particular pharmacological interest. AChE and BChE are therapeutic targets for Alzheimer's disease, while amylase and glucosidase are key enzymes in diabetes control. The search for natural inhibitors of these enzymes is therefore an important strategy for the development of new therapeutic agents. In this study, the extracts of the three species of the genus *Hypericum* were evaluated for their ability to inhibit key enzymes such as AChE, BChE, tyrosinase, amylase, and glucosidase. Results, reported in Table 4, show that for AChE inhibition, the *H. scabrum* ethanol/water extract showed the best activity (2.91 ± 0.09 mg GALAE/g), followed by *H. lysimachioides* ethanol/water (2.81 ± 0.09 mg GALAE/g). The aqueous extract of *H. lysimachioides* displayed significantly lower activity (1.04 ± 0.06 mg GALAE/g), and the aqueous extract of *H. scabrum* showed the lowest value (0.54 ± 0.01 mg GALAE/g). No activity was observed for *H. uniglandulosum*—ethanol/water and water extracts. For BChE, the *H. scabrum* aqueous extract showed the best inhibiting activity, with a value of 1.13 ± 0.03 mg GALAE/g, followed by the *H. uniglandulosum* hydroalcoholic extract (1.05 ± 0.11 mg GALAE/g), *H. scabrum* and *H. lysimachioides* hydroalcoholic extracts (0.99 ± 0.02 mg GALAE/g and 0.43 ± 0.04 mg GALAE/g, respectively), and the *H. uniglandulosum* aqueous extract, with a value of 0.23 ± 0.04 mg GALAE/g. No inhibition against BChE was observed for the *H. lysimachioides* aqueous extract. Tyrosinase inhibition was highest for *H. lysimachioides* with ethanol/water (67.39 ± 0.52 mg KAE/g), while the aqueous extracts showed significantly lower activity (13.29 ± 1.28 mg KAE/g). The hydroalcoholic extract of *H. scabrum* reported a value of 61.19 ± 3.49 mg KAE/g, higher than its water extract (15.71 ± 1.07 mg KAE/g). The *H. uniglandulosum*—ethanol/water extract showed a tyrosinase inhibition value of 48.87 ± 0.10 mg KAE/g. Its aqueous extract showed the lowest activity, with a value of 4.10 ± 0.29 mg KAE/g. Inhibitory activity against amylase was quite low in all species and solvents, with values ranging between 0.04 ± 0.01 mmol ACAE/g for all water extracts and 0.24 mmol ACAE/g for the hydroalcoholic extract of *H. uniglandulosum*. However, for glucosidase, the best activity was observed for hydroalcoholic extracts of *H. scabrum* and *H. lysimachioides*, with values of 3.35 ± 0.03 mmol ACAE/g and 3.30 ± 0.01 mmol ACAE/g, respectively. *H. uniglandulosum* extracts were the weakest against glucosidase, with its aqueous extract exhibiting a value of 0.72 ± 0.06 mmol ACAE/g. There are few or no studies in the literature on the enzymatic inhibition ability of these three species of *Hypericum*, with results often not comparable with our study, mainly due to different methodologies used to evaluate this capacity. A study on extracts of *H. scabrum* found that the essential oils exhibited high inhibitory effects on AChE and BChE, surpassing the standard compound galantamine. Additionally, this species demonstrated significant inhibitory activity on tyrosinase and moderate inhibitory effects on elastase (Akdeniz et al. 2022). *H. lysimachioides* and *H. uniglandulosum* have not been extensively studied for their enzyme inhibitory capacity. However, many studies show that numerous *Hypericum* species possess good enzymatic inhibition capabilities. For example, *Hypericum laricifolium* Juss. exhibited notable inhibition of AChE activity, with IC50 values ranging from 432.74 to over 1500.00 μg of dry extract per milliliter (Božin et al. 2013). *Hypericum humifusum* and *Hypericum perfoliatum* also demonstrated inhibitory potential against AChE and key enzymes related to type 2 diabetes (Béjaoui et al. 2017). Another study on extracts of *H. laricifolium* highlighted significant inhibitory activities against α‐glucosidase (97.2% at 500 μg/mL) and aldose reductase (56.9% at 500 μg/mL), enzymes linked to diabetes (Guillen Quispe et al. 2017).

**TABLE 4:** Enzyme inhibitory properties of the tested extracts.**

Solvents	Species	AChE (mg GALAE/g)	BChE (mg GALAE/g)	Tyrosinase (mg KAE/g)	Amylase (mmol ACAE/g)	Glucosidase (mmol ACAE/g)
Ethanol/Water (70%)	*H. lysimachioides*	2.81 ± 0.09^a^	0.43 ± 0.04^b^	67.39 ± 0.52^a^	0.18 ± 0.01^c^	3.30 ± 0.01^a^
	*H. scabrum*	2.91 ± 0.09^a^	0.99 ± 0.02^a^	61.19 ± 3.49^b^	0.19 ± 0.01^b^	3.35 ± 0.03^a^
	*H. uniglandulosum*	na	1.05 ± 0.11^a^	48.87 ± 0.10^c^	0.24 ± 0.01^a^	1.95 ± 0.22^c^
Water	*H. lysimachioides*	1.04 ± 0.06^b^	na	13.29 ± 1.28^d^	0.04 ± 0.01^d^	2.85 ± 0.04^b^
	*H. scabrum*	0.54 ± 0.01^c^	1.13 ± 0.03^a^	15.71 ± 1.07^d^	0.04 ± 0.01^d^	2.95 ± 0.03^b^
	*H. uniglandulosum*	na	0.23 ± 0.04^c^	4.10 ± 0.29^e^	0.04 ± 0.01^d^	0.72 ± 0.06^d^

*Note:* Different letters indicate significant differences between the tested extracts (*p* < 0.05). Abbreviations: ACAE, acarbose equivalent; GALAE, galantamine equivalent; KAE, Kojic acid equivalent; na, not active.

** Values are reported as mean ± SD of three parallel measurements.

### 3.5 Neuroprotective and Neuromodulator Effects

Neuroinflammation has been suggested to be implicated in the onset of neuropsychiatric disorders, including depression, (Capuron and Castanon 2017) and in this context, an inflammatory stimulus like LPS has been reported to increase the burden of inflammation and oxidative stress and, in parallel, the monoamine neurotransmitter turnover, in mouse brain (de Monchaux Oliveira et al. 2023). By contrast, the pretreatment with saffron ( *Crocus sativus* L.), a well‐known antidepressant plant (Dobrek and Głowacka 2023), was able to revert the mood alteration. Also, *H. perforatum* extracts have long been reported to be effective as antidepressants (Dobrek and Głowacka 2023). On the other hand, the antidepressant efficacy of the three *Hypericum* species that are the object of the present study is still a matter of debate, with only a few papers reporting potential antidepressant and antianxiety effects from the phytochemicals of *H. scabrum* (Ganji et al. 2017; Ma et al. 2021).

Therefore, in the present study, we investigated the effects of ethanol and water extracts (50–100 μg/mL) from *H. scabrum* , *H. lysimachioides*, and *H. uniglandulosum* on the gene expression of COX‐2 and NOS‐2, as indexes of inflammation, and SERT and NET, as biomarkers of serotonin and norepinephrine signaling, respectively, in isolated mouse brain specimens exposed to LPS, a validated experimental model of neuroinflammation (Chichiriccò et al. 2019).

**FIGURE 4:**
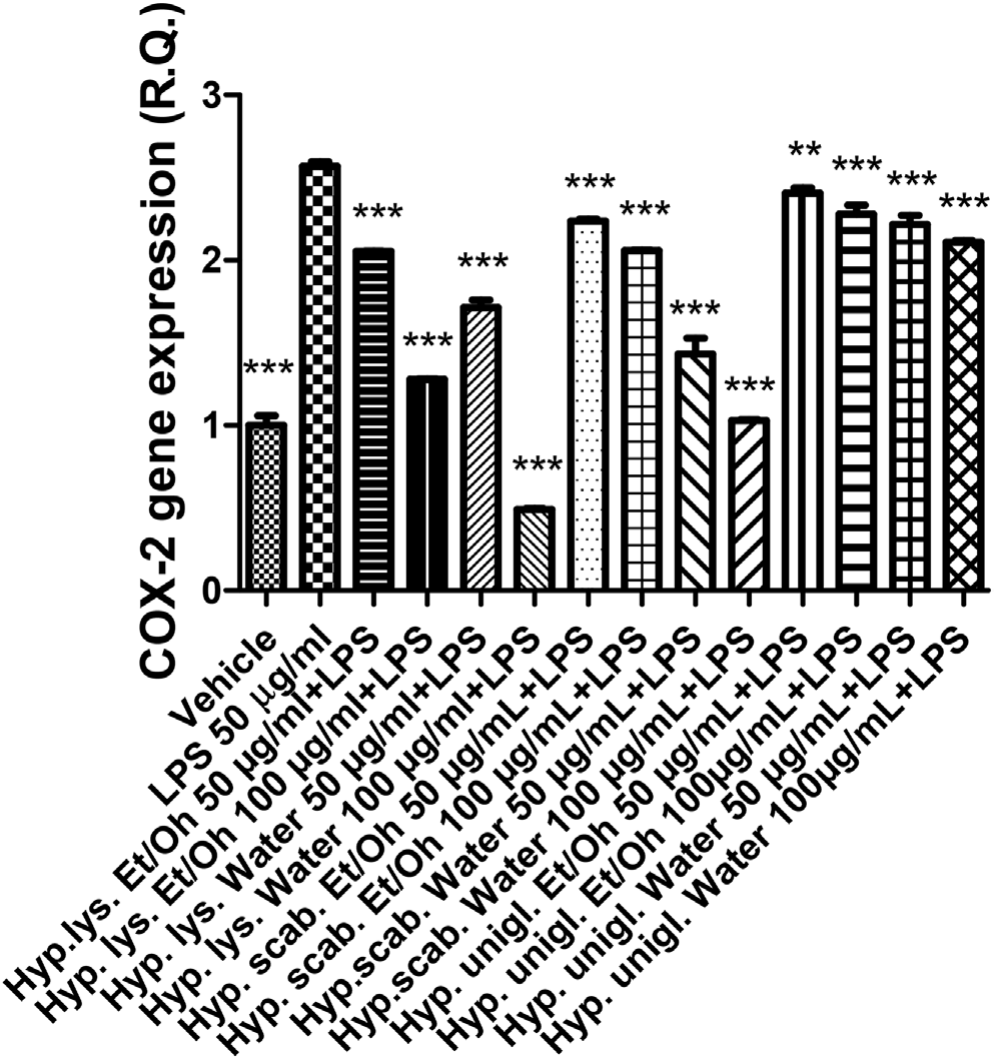
Inhibitory effects induced by the water and ethanol extracts (50–100 μg/mL) from the three *Hypericum* species on LPS‐induced COX‐2 gene expression in isolated mouse cortex. ANOVA, *p* < 0.0001; ***p < 0.001 vs. vehicle group.

**FIGURE 5:**
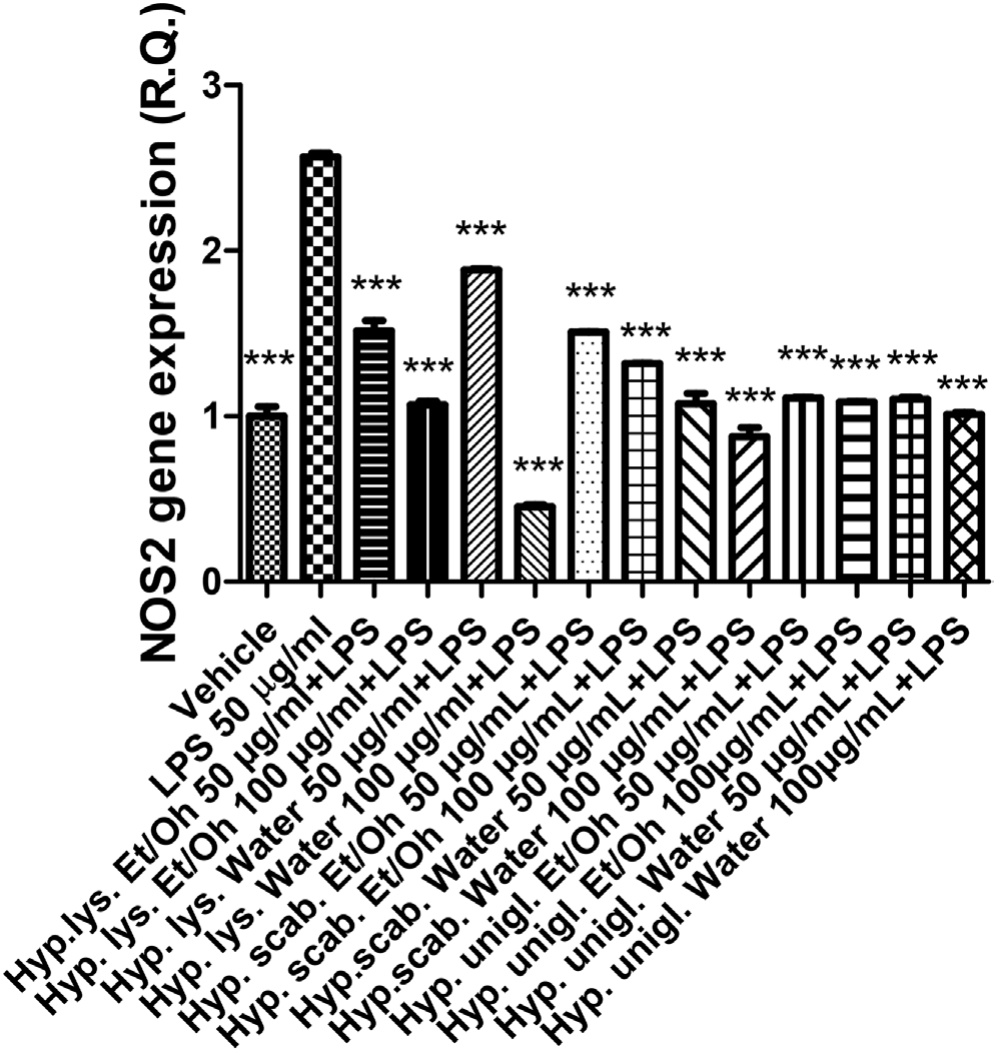
Inhibitory effects induced by the water and ethanol extracts (50–100 μg/mL) from the three *Hypericum* species on LPS‐induced NOS‐2 gene expression in isolated mouse cortex. ANOVA, *p* < 0.0001; ***p < 0.001 vs. vehicle group.

**FIGURE 6:**
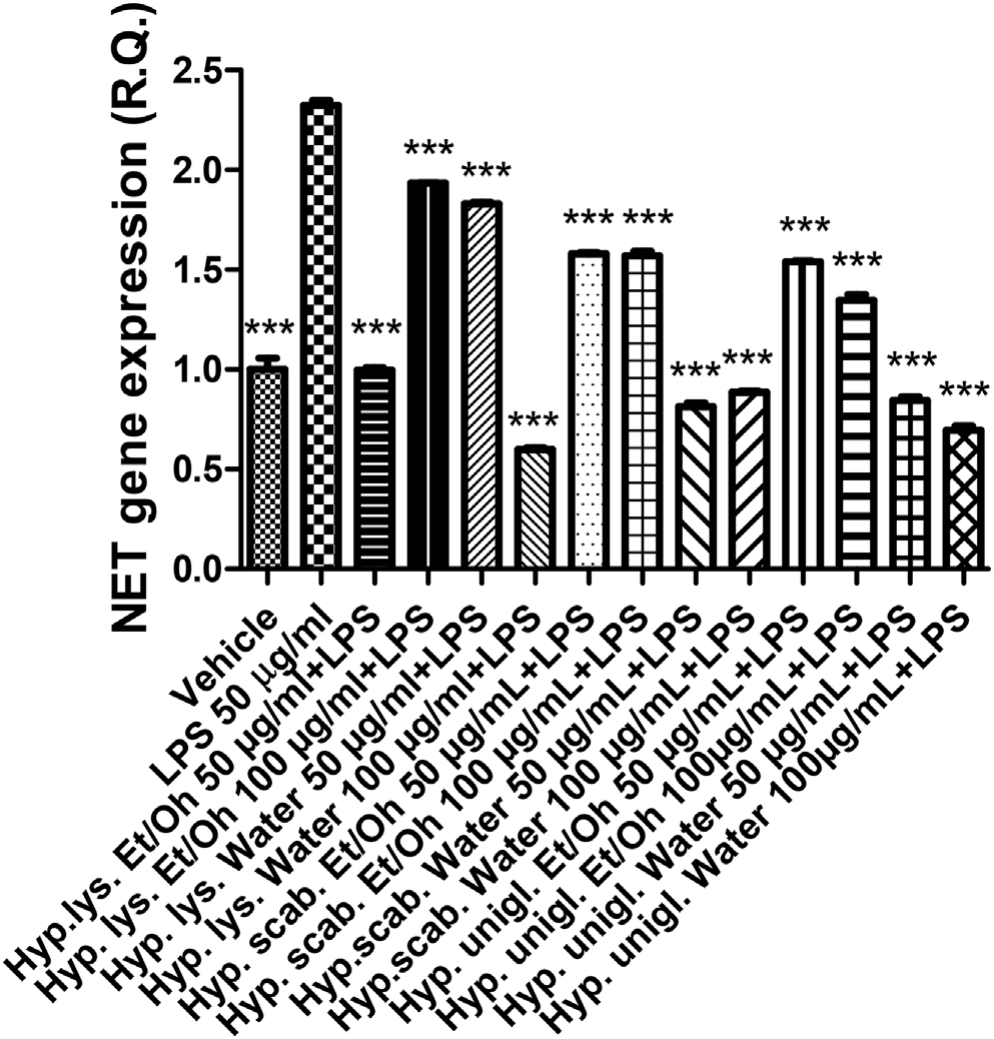
Inhibitory effects induced by the water and ethanol extracts (50–100 μg/mL) from the three *Hypericum* species on LPS‐induced NET gene expression in isolated mouse cortex. ANOVA, *p* < 0.0001; ***p < 0.001 vs. vehicle group.

**FIGURE 7:**
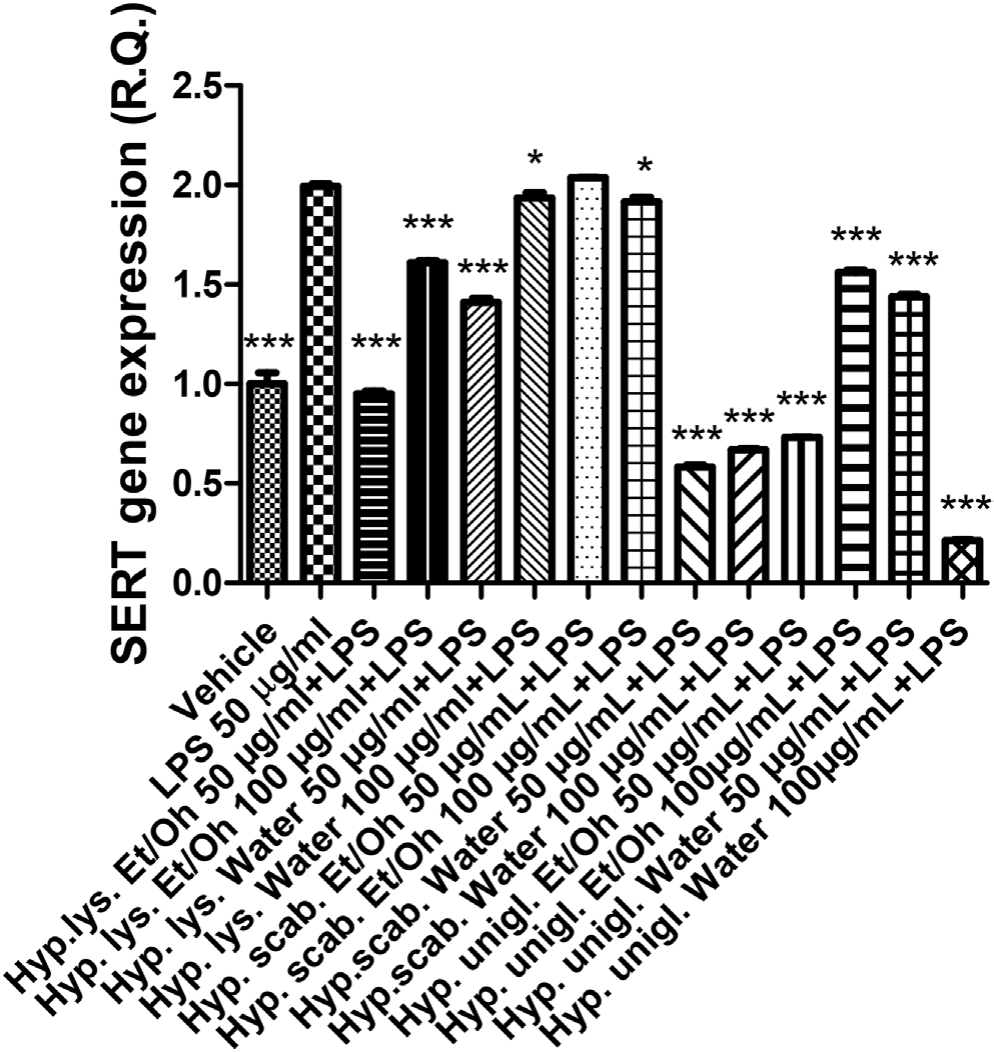
Inhibitory effects induced by the water and ethanol extracts (50–100 μg/mL) from the three *Hypericum* species on LPS‐induced SERT gene expression in isolated mouse cortex. ANOVA, *p* < 0.0001; ***p < 0.001 vs. vehicle group.

The LPS stimulus caused a significant increase in the gene expression of COX‐2 and NOS‐2 (Figures 4, 5), indicating a stimulation of inflammation and nitrosative stress, respectively. The increase in both enzymes has been related to the onset of depression (Beheshti et al. 2020; He et al. 2022). In parallel, the inflammatory stimulus led to an increase in both SERT and NET gene expressions (Figures 6, 7) that could account for a reduction of serotonin and norepinephrine release from presynaptic endings, whereas the inhibition of both transporters is a classical pharmacological target for antidepressant therapy (Hamon and Blier 2013).

All extracts were effective in preventing the LPS stimulation of COX‐2, NOS‐2, SERT, and NET; thus, suggesting both antineuroinflammatory and stimulating effects on monoamine release that pose the basis for potential applications of *H. scabrum* , *H. lysimachioides*, and *H. uniglandulosum* bioactive extracts as ingredients in innovative antidepressant agents.

Considering the richness in specialized metabolites of the investigated phytocomplexes, different concomitant mechanisms could be at the basis of the observed anti‐inflammatory effects. For instance, the presence of flavonoids, such as catechins, could explain, albeit partially, the inhibition of COX‐2 and NOS‐2 gene expression (Recinella et al. 2022). This is of particular relevance for *H. scabrum and H. lysimachioides* extracts that, besides being the most effective as anti‐inflammatory agents, were also the most effective as antiradical and enzyme inhibition agents (Tables 3, 4). These extracts also displayed the highest content in total phenols and flavonoids (Table 2), and in this regard, it is sensitive to highlight how also enzyme inhibition and scavenging/reducing effects are deeply related to the extracts' content in total phenols and flavonoids (di Giacomo et al. 2021). Regarding the NET and SERT gene expression, *H. uniglandulosum* extracts showed the highest potency as inhibitors. Although containing hypericins that could act as monoamine oxidase inhibitors (Tusevski et al. 2024), it is questionable that these compounds could mediate the observed inhibitory effects on monoamine transporter inhibition (Butterweck 2003). Additionally, considering the lowest content in total phenols and flavonoids shown by *H. uniglandulosum* extracts, it is more conceivable that other unidentified phytocompounds present in the extracts could be responsible for such effects.

### 3.6 Further Insights on Active Compounds: Targeting Cancer

**FIGURE 8:**
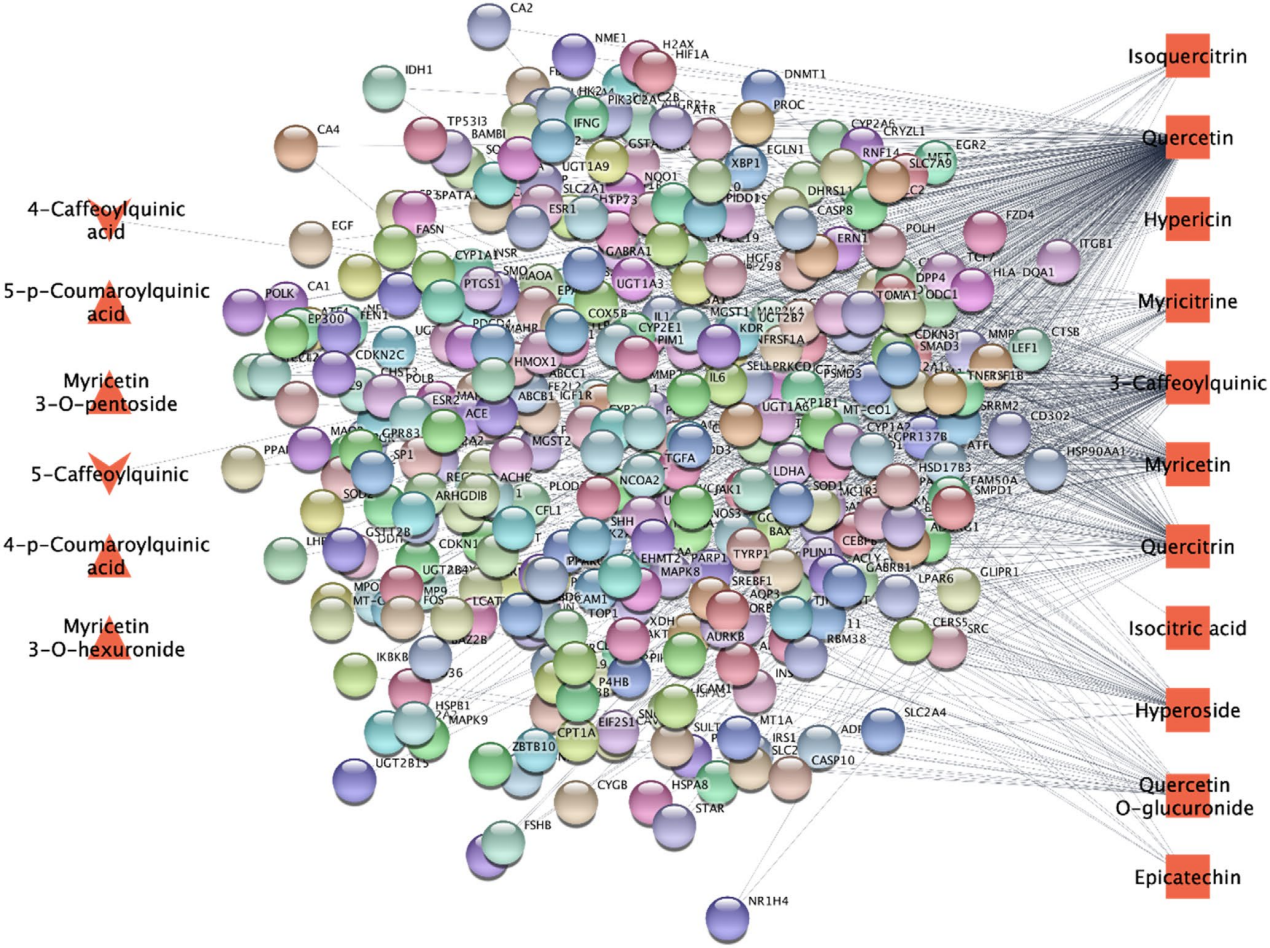
Target analysis of *Hypericum spp*. compounds.

Following the identification of genes associated with phytochemicals derived from the tested *Hypericum* species, a Venn diagram was employed to investigate the relationships between these genes and those specific to anxiety disorders, depression, and neuroinflammation. The comparative analysis encompassed 696 genes associated with the tested *Hypericum* compounds with a degree of bigger than 2 (Figure 8). In the case of depression, 39 genes were identified using the STRING V12.0 database. After validation, 136 of these genes were found to interact, forming a total of 152 edges. The maximal clique centrality (MCC) technique, implemented via the CytoHubba plugin, revealed key hub genes such as TP53, HIF1A, HSPA5, and BCL2. In the analysis of anxiety disorders, nine genes were identified using the STRING V12.0 database. After validation, nine of these genes were found to interact, forming a total of 17 edges. The MCC technique, implemented via the CytoHubba plugin, revealed key hub genes such as INS, APOE, APP, and ACE2. In the analysis of neuroinflammation, 35 genes were identified using the STRING V12.0 database. After validation, 35 of these genes were found to interact, forming a total of 407 edges. The MCC technique, implemented via the CytoHubba plugin, revealed key hub genes such as IFNG, IL1B, IL6, and STAT3.

**FIGURE 9:**
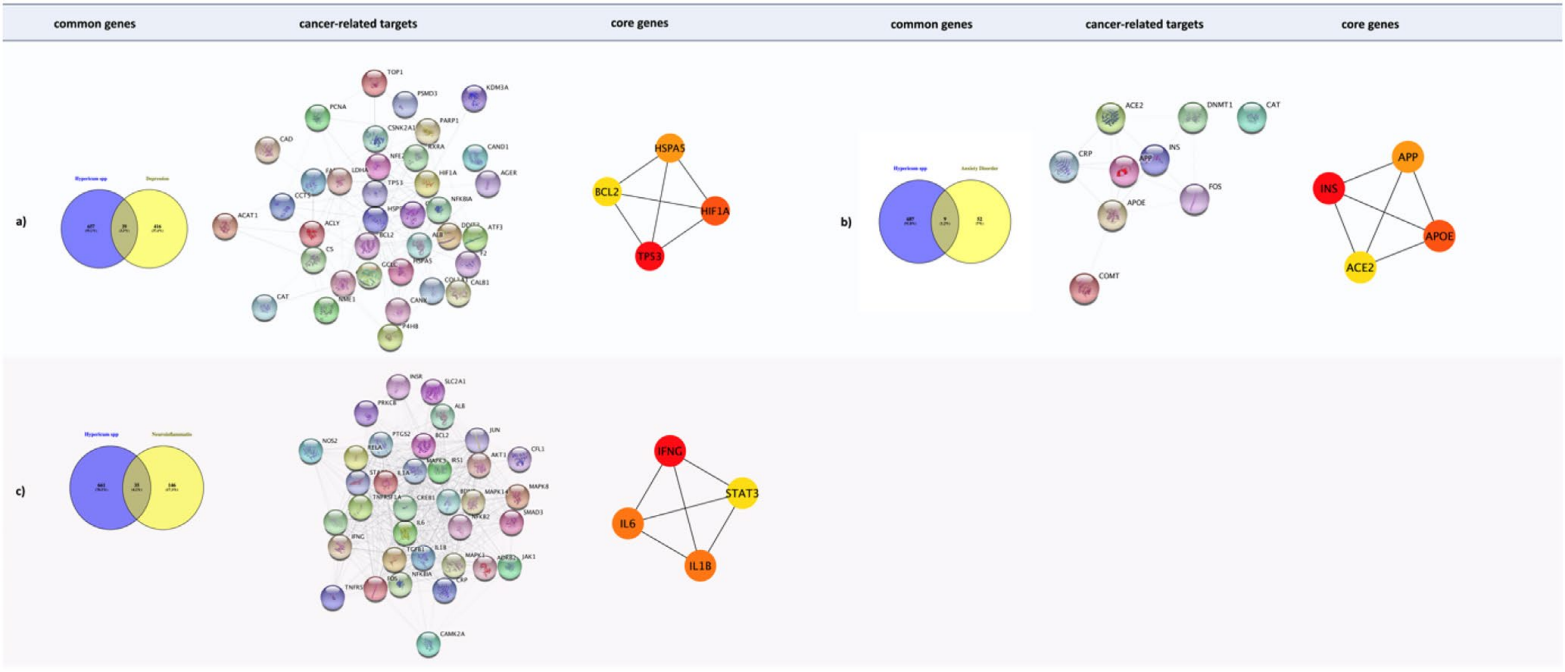
Target analysis of disease‐related genes: (a) depression, (b) anxiety disorders, and (c) neuroinflammation.

The gene associations were retrieved from the CTD and GeneCards databases (Figure 9). The Venn diagram illustrates the overlap between the gene sets associated with these phytochemicals and the selected disease types. Based on the number of edges, quercetin and 3‐caffeoyquinic molecules were identified to have potentially significant associations (Figure 8).

### 3.7 KEGG Analysis of Cancer‐Related Molecular Pathways of *Hypericum* Compounds

**FIGURE 10:**
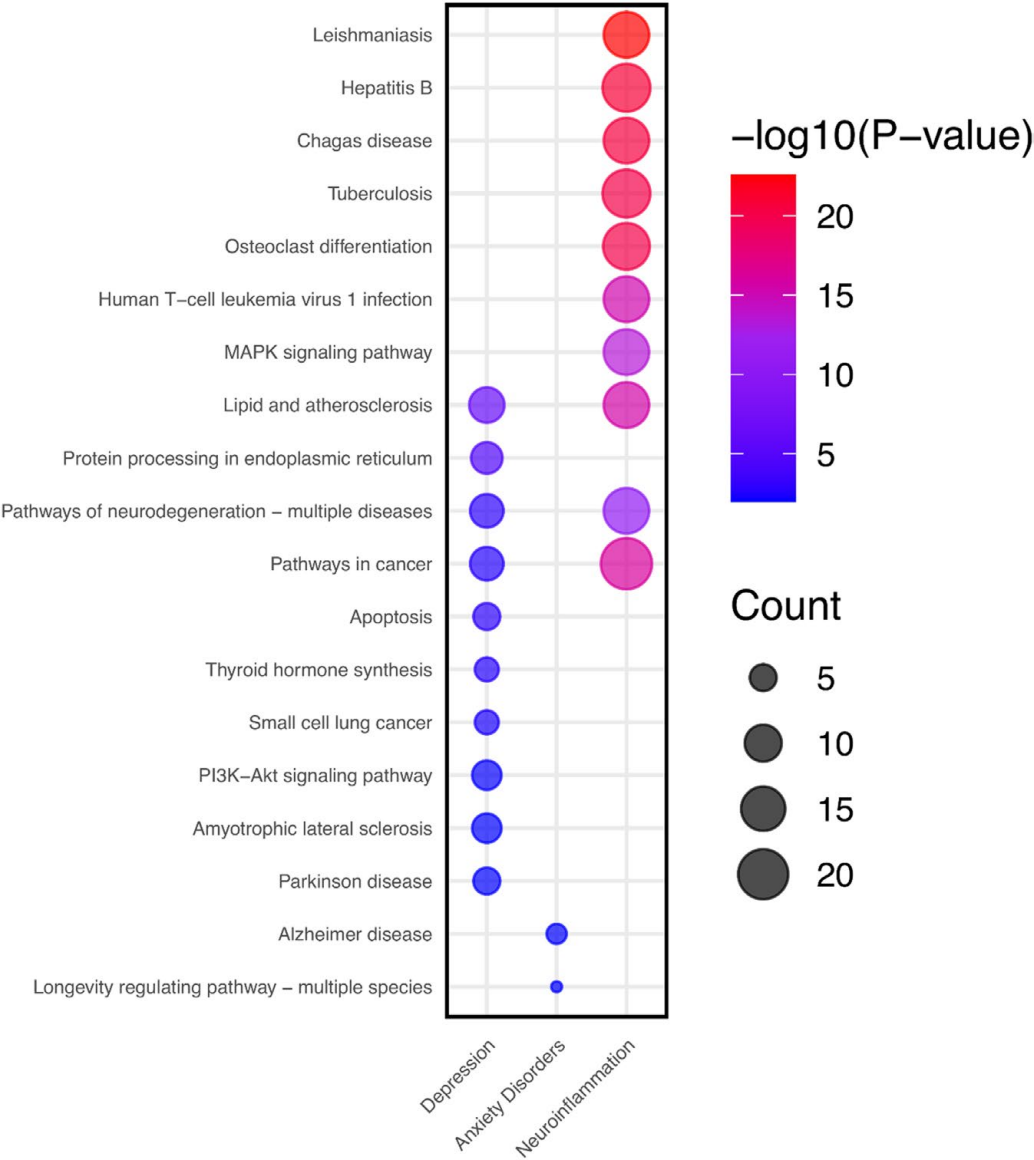
KEGG pathway enrichment analysis of disease‐related terms.

A target pathway enrichment study was performed using the DAVID 6.8 tool to investigate the possible biological effects of the tested *Hypericum* components on depression, anxiety disorder, and neuroinflammation. The study identified 23 pathways associated with depression, 2 pathways associated with anxiety disorder, and 153 pathways associated with neuroinflammation, based on statistical significance (*p* < 0.05). The analysis of biological pathways in depression, anxiety disorder, and neuroinflammation reveals both nondistinct and shared biological mechanisms for each disease type. In the context of depression, notable enrichment was observed in pathways such as lipid and atherosclerosis, protein processing in the endoplasmic reticulum, pathways of neurodegeneration—multiple diseases, and pathways in cancer. Among these, the lipid and atherosclerosis pathway is a shared feature between depression and neuroinflammation, but it does not show enrichment in anxiety disorders. This finding may reflect the existence of shared biological mechanisms between depression and neuroinflammation. In the context of neuroinflammation, pathways pertaining to infection and immune response, including those related to cancer, hepatitis B, tuberculosis, osteoclast differentiation, and leishmaniasis, exhibit notable enrichment. Furthermore, the MAPK and PI3K‐Akt signaling pathways are significantly enriched in neuroinflammation, playing a pivotal role in cellular signaling processes. With regard to anxiety disorders, a slight enrichment was observed in pathways such as Alzheimer's disease and the longevity regulating pathway—multiple species. However, the degree of enrichment in these pathways is less pronounced in comparison to those associated with depression and neuroinflammation. These findings emphasize the significance of the lipid and atherosclerosis pathway, which is shared between depression and neuroinflammation, while also underscoring the substantial enrichment of the MAPK and PI3K‐Akt pathways, particularly in the context of neuroinflammation. These pathways may be regarded as potential therapeutic targets. Overall, the data reveal distinctive biological mechanisms for each condition, as well as shared pathways (Figure 10).

**FIGURE 11:**
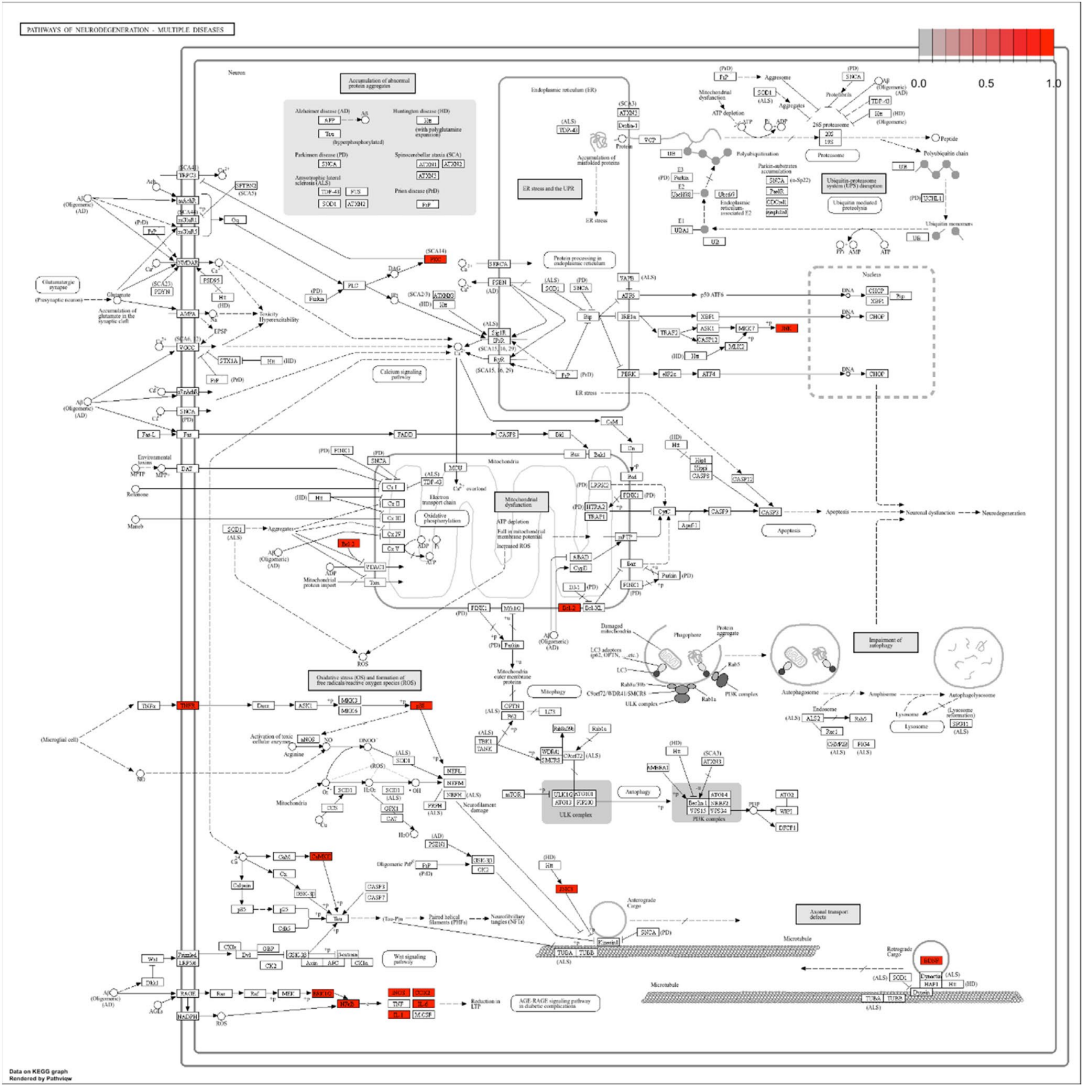
Pathways of neurodegeneration—multiple diseases pathway enrichment analysis in neuroinflammation, highlighting *Hypericum spp*. compound targets.

The pathway enrichment analysis performed on the pathways of neurodegeneration—multiple diseases pathway shows that 16 genes play a significant role in understanding the relationship of this pathway with neurodegenerative processes. These genes include NOS2, PRKCB, BDNF, CAMK2A, PTGS2, MAPK14, TNFRSF1B, RELA, TNFRSF1A, IL1A, IL6, MAPK8, IL1B, BCL2, MAPK1, and MAPK3. This pathway includes genes that are critical for signaling processes in the pathophysiology of neurodegenerative diseases. For example, IL‐6, IL1B, and PTGS2 are particularly important for their role in inflammatory responses that are central to neuroinflammation in neurodegenerative diseases. IL‐6 is a pro‐inflammatory cytokine that influences immune responses and has been implicated in exacerbating neuroinflammation, contributing to the progression of neurodegenerative diseases. IL1B, another key cytokine, mediates inflammatory signaling and is involved in promoting cellular responses that can lead to neurotoxicity and tissue damage in the brain. PTGS2 (also known as COX‐2) is an enzyme that plays a key role in the synthesis of prostaglandins, lipid compounds that mediate inflammation and pain; increased PTGS2 expression is often associated with chronic neuroinflammatory conditions. Other genes in this pathway also contribute to the pathological processes of neurodegeneration. For example, NOS2 and PTGS2 regulate inflammatory responses, while BDNF and CAMK2A are associated with neuronal survival and plasticity. Members of the MAPK family, including MAPK14, MAPK8, MAPK1, and MAPK3, are essential for intracellular signaling and stress responses. In addition, tumor necrosis factor receptors such as TNFRSF1A and TNFRSF1B regulate cell death and survival, which may influence the progression of neurodegenerative diseases. The enrichment of these genes within this pathway underscores the convergence of biological processes like inflammation, cell death, synaptic plasticity, and signal transduction in the development of neurodegenerative diseases. Targeting these processes, particularly focusing on inflammatory mediators like IL‐6, IL1B, and PTGS2, holds promise for developing novel therapeutic strategies to treat neurodegenerative diseases (Figure 11).

### 3.8 Evaluating Docking Outcomes: Ligand Binding Energies and Interaction Profiles

**FIGURE 12:**
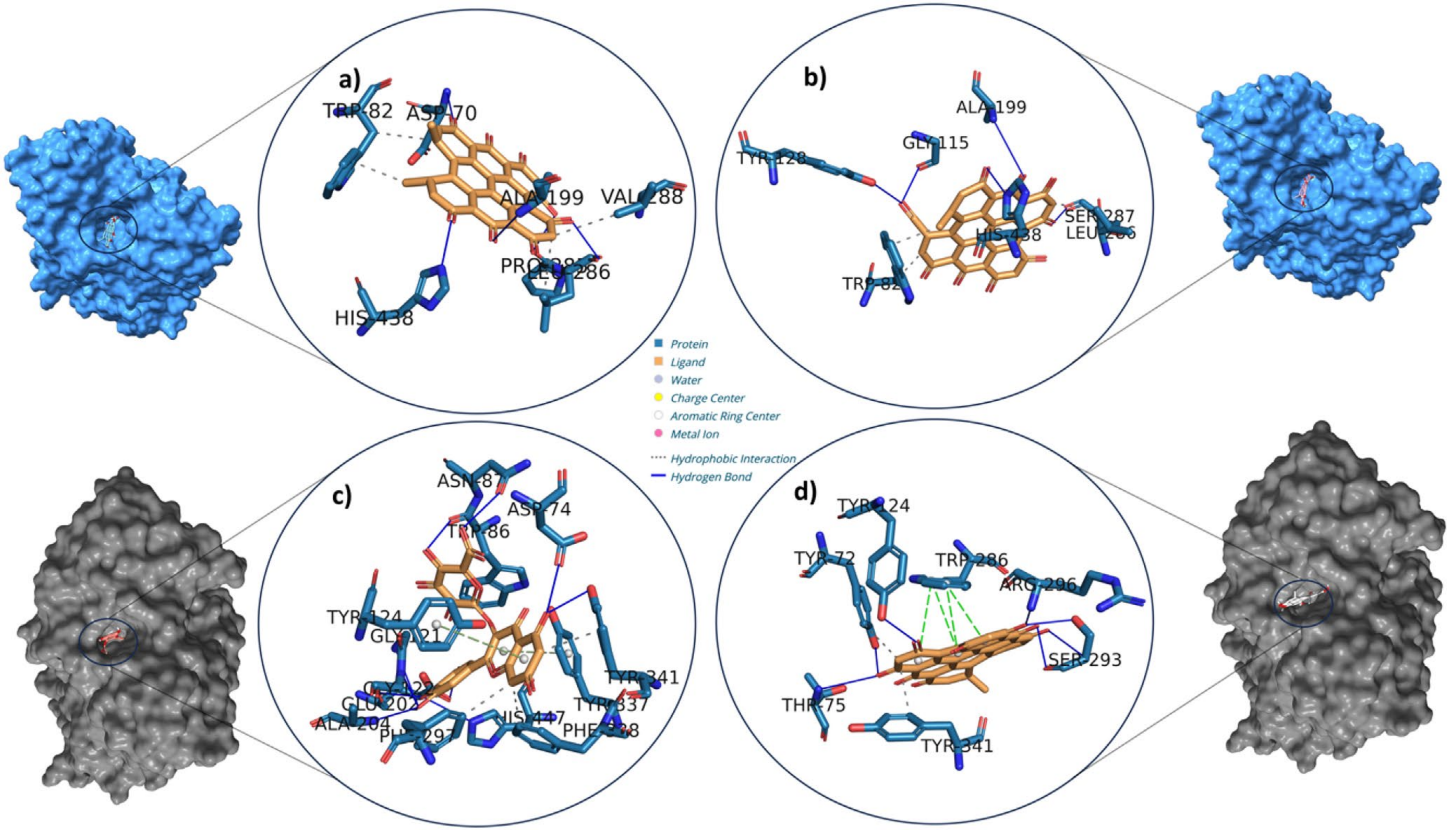
Binding interactions of enzymes with compounds showing the best binding energy: (a) interaction between BChE and hypericin, (b) interaction between BChE and pseudohypericin, (c) interaction between AChE and myricetin 3‐*O*‐hexuronide, and (d) interaction between AChE and hypericin.

This study employed a comprehensive evaluation approach to examine the antidisease properties of compounds identified in the tested extracts against standard enzymes and disease‐associated proteins and enzymes. The coordinates and grid dimensions employed in the analyses are provided in Table S1 for reference. The chemical profiling revealed a substantial number of bioactive compounds, including myricetin 3‐*O*‐hexuronide, myricetin 3‐*O*‐hexoside, quercetin *O*‐glucuronide, quercitrin, epicatechin, myricitrin, myricetin, 3‐*O*‐caffeoylquinic acid, and 5‐*O*‐caffeoylquinic acid, hypericin, 4‐*p*‐coumaroylquinic acid, hyperoside, isoquercitrin, 4‐*O*‐caffeoylquinic acid, quercetin, isocitric acid, myricetin 3‐*O*‐pentoside, 5‐*p*‐coumaroylquinic acid, and pseudohypericin. The standard enzymes AChE, BChE, tyrosinase, amylase, and glucosidase were analyzed in conjunction with key proteins derived from neuroblastoma, hepatocellular carcinoma, and lung adenocarcinoma. The primary objective was to explore the anticancer activities of these phytochemicals in relation to cancer‐related proteins and standard enzymes. Table 1 highlights compounds with binding energies below −9 kcal/mol, while Table S2 lists those with binding energies above this threshold. The docking results demonstrated a range of binding energies from −10.8 to −4.4 kcal/mol and RMSD from 1.2 to 0.1 (Table S2; Table 5; Figure 12).

**TABLE 5:** The docking score (kcal/mol) and interacting residues of the enzyme and protein.

Compound	Target	PDB ID	Binding energy	RMSD	Interaction		Binding site
					Type	Number	
Myricetin 3‐*O*‐hexuronide	*BChE*	3djy	−10.4	1.0	H‐bond	8	THR A:120, TYR A:128, TYR A:128, GLU A:197, ALA A:199, TYR A:332, TYR A:332, HIS A:438
Myricetin 3‐*O*‐hexoside	*BChE*	3djy	−9.9	0.1	H‐bond	8	ASP A:70, ASP A:70, TRP A:82, GLY A:115, GLY A:115, TYR A:128, GLU A:197, HIS A:438
Quercetin *O*‐glucuronide	*BChE*	3djy	−10.2	0.8	H‐bond	7	ASP A:70, GLY A:115, GLY A:116, GLY A:117, GLU A:197, ALA A:199, ALA A:199
Quercitrin	*BChE*	3djy	−10.4	1.1	H‐bond	12	ASP A:70, ASP A:70, GLY A:78, GLY A:115, GLY A:117, TYR A:128, TYR A:128, TYR A:128, GLU A:197, SER A:287, TYR A:332, HIS A:438
Epicatechin	*BChE*	3djy	−9.2	0.2	H‐bond	5	ASP A:70, ASP A:70, ASP A:70, TRP A:82, TYR A:332
Mricitrin	*BChE*	3djy	−10.4	1.0	H‐bond	11	ASP A:70, TRP A:82, GLY A:115, GLY A:116, GLY A:117, TYR A:128, TYR A:128, GLU A:197, ALA A:199, TYR A:332, TYR A:332
Myricetin	*BChE*	3djy	−9.3	1.0	H‐bond	5	ASP A:70, ASP A:70, ASP A:70, ASN A:83, TYR A:332
Hypericin	*BChE*	3djy	−12.2	1.0	H‐bond	5	ASP A:70, ALA A:199, PRO A:285, LEU A:286, HIS A:438
Hyperoside	*BChE*	3djy	−10.4	0.7	H‐bond	10	ASP A:70, TRP A:82, GLY A:115, GLY A:115, TYR A:128, TYR A:128, ALA A:199, ALA A:199, TYR A:332, HIS A:438
Isoquercitrin	*BChE*	3djy	−10.4	1.1	H‐bond	11	GLY A:115, GLY A:115, GLY A:116, THR A:120, TYR A:128, GLU A:197, GLU A:197, ALA A:199, TYR A:332, TYR A:332, HIS A:438
Quercetin	*BChE*	3djy	−9.4	0.4	H‐bond	4	ASP A:70, ASP A:70, ASN A:83, TYR A:332
Myricetin 3‐*O*‐pentoside	*BChE*	3djy	−9.5	3.6	H‐bond	9	ASN A:68, ASP A:70, GLY A:115, GLY A:115, GLY A:116, GLY A:117, GLU A:197, ALA A:199, HIS A:438
Pseudohypericin	*BChE*	3djy	−11.6	0.3	H‐bond	5	GLY A:115, TYR A:128, ALA A:199, SER A:287, HIS A:438
Epicatechin	*Amylase*	2qv4	−9.0	1.0	H‐bond	6	GLN A:63, ARG A:195, ARG A:195, ASP A:197, ASP A:300, HIS A:305
Myricitrin	*Amylase*	2qv4	−9.3	0.5	H‐bond	11	THR A:163, ARG A:195, ARG A:195, ASP A:197, LYS A:200, HIS A:201, HIS A:299, ASP A:300, ASP A:300, ASP A:300, HIS A:305
Myricetin	*Amylase*	2qv4	−9.3	1.0	H‐bond	5	GLN A:63, ARG A:195, HIS A:299, ASP A:300, ASP A:300
Hypericin	*Amylase*	2qv4	−10.8	0.1	H‐bond	5	TRP A:59, GLN A:63, HIS A:101, ALA A:198, GLU A:233
Quercetin	*Amylase*	2qv4	−9.2	0.1	H‐bond	6	TYR A:62, GLN A:63, ARG A:195, ASP A:197, ASP A:197, HIS A:299
Myricetin 3‐*O*‐pentoside	*Amylase*	2qv4	−9.0	1.1	H‐bond	8	GLN A:63, GLN A:63, ASP A:197, HIS A:299, ASP A:300, ASP A:300, HIS A:305, HIS A:305
Pseudohypericin	*Amylase*	2qv4	−10.1	0.7	H‐bond	7	TRP A:59, GLN A:63, HIS A:101, ASP A:197, ALA A:198, GLU A:233, HIS A:305
Hypericin	*Glucosidase*	3w37	−9.0	0.1	H‐bond	5	ASP A:362, ASP A:362, LEU A:369, ARG A:412, ARG A:412
Myricetin 3‐*O*‐hexuronide	*AChE*	2y2v	−10.8	0.4	H‐bond	10	ASP A:74, TRP A:86, ASN A:87, GLY A:121, GLY A:122, GLU A:202, GLU A:202, ALA A:204, TYR A:341, HIS A:447
Myricetin 3‐*O*‐hexoside	*AChE*	2y2v	−9.8	0.1	H‐bond	12	TRP A:86, GLY A:121, GLY A:122, TYR A:124, TYR A:133, TYR A:133, GLU A:202, GLU A:202, PHE A:295, ARG A:296, TYR A:341, HIS A:447
Quercetin *O*‐glucuronide	*AChE*	2y2v	−10.4	0.2	H‐bond	16	TYR A:72, ASP A:74, ASP A:74, TRP A:86, GLY A:121, GLY A:122, TYR A:124, SER A:125, GLY A:126, GLU A:202, ALA A:204, ALA A:204, PHE A:295, ARG A:296, TYR A:341, HIS A:447
Quercitrin	*AChE*	2y2v	−10.1	0.8	H‐bond	11	GLY A:121, GLY A:122, TYR A:124, SER A:125, SER A:125, GLU A:202, ALA A:204, PHE A:295, ARG A:296, TYR A:341, HIS A:447
Epicatechin	*AChE*	2y2v	−9.6	1.1	H‐bond	4	TYR A:72, TYR A:72, ASP A:74, SER A:125
Myricitrin	*AChE*	2y2v	−10.2	0.9	H‐bond	10	TRP A:86, TRP A:86, GLY A:121, GLY A:122, TYR A:124, TYR A:124, PHE A:295, ARG A:296, TYR A:341, HIS A:447
Myricetin	*AChE*	2y2v	−10.2	1.0	H‐bond	6	TYR A:72, ASP A:74, ASP A:74, ASN A:87, TYR A:133, GLU A:202
3‐Caffeoylquinic	*AChE*	2y2v	−9.4	0.9	H‐bond	8	GLY A:121, GLY A:122, GLU A:202, ALA A:204, PHE A:295, ARG A:296, ARG A:296, HIS A:447
5‐Caffeoylquinic	*AChE*	2y2v	−9.3	1.0	H‐bond	2	SER A:125, PHE A:295
Hypericin	*AChE*	2y2v	−11.4	1.1	H‐bond	7	TYR A:72, THR A:75, TYR A:124, SER A:293, SER A:293, SER A:293, ARG A:296
4‐*p*‐Coumaroylquinic acid	*AChE*	2y2v	−9.2	0.4	H‐bond	1	TYR A:341
Hyperoside	*AChE*	2y2v	−10.0	1.1	H‐bond	12	ASP A:74, TRP A:86, ASN A:87, GLY A:121, GLY A:122, TYR A:124, TYR A:133, PHE A:295, ARG A:296, ARG A:296, TYR A:341, HIS A:447
4‐Caffeoylquinic acid	*AChE*	2y2v	−9.2	0.9	H‐bond	9	GLN A:71, ASP A:74, ASN A:87, GLY A:121, GLY A:122, GLU A:202, ALA A:204, ALA A:204, HIS A:447
Quercetin	*AChE*	2y2v	−9.5	6.0	H‐bond	5	TYR A:72, ASP A:74, GLY A:120, TYR A:133, GLU A:202
Myricetin 3‐*O*‐pentoside	*AChE*	2y2v	−10.5	0.7	H‐bond	6	TRP A:86, GLY A:120, TYR A:124, GLU A:202, GLU A:202, HIS A:447
5‐*p*‐Coumaroylquinic acid	*AChE*	2y2v	−9.5	0.2	H‐bond	5	TRP A:86, SER A:125, PHE A:295, TYR A:341, TYR A:341
Pseudohypericin	*AChE*	2y2v	−10.5	9.2	H‐bond	7	TYR A:72, TYR A:72, TYR A:124, SER A:293, SER A:293, SER A:293, ARG A:296
Myricetin 3‐*O*‐hexuronide	PTGS2	5F19	−9.3	0.8	H‐bond	9	ASN A:34, ASN A:34, CYS A:36, CYS A:47, CYS A:47, SER A:49, SER A:49, GLY A:135, ASP A:157
Epicatechin	PTGS2	5F19	−9.0	9.9	H‐bond	4	ASN A:34, GLY A:45, CYS A:47, GLN A:461
Myricitrin	PTGS2	5F19	−9.2	0.5	H‐bond	7	ASN A:34, ASN A:34, CYS A:36, CYS A:47, SER A:49, SER A:49, TYR A:130
Myricetin	PTGS2	5F19	−9.1	None	H‐bond	5	ASN A:34, HIS A:39, CYS A:47, GLY A:135, GLN A:461
Hypericin	PTGS2	5F19	−9.9	9.3	H‐bond	5	CYS A:47, SER A:49, GLY A:135, GLY A:135, ASP A:157
Quercetin	PTGS2	5F19	−9.5	None	H‐bond	6	HIS A:39, GLY A:45, CYS A:47, CYS A:47, GLU A:465, GLU A:465
Pseudohypericin	PTGS2	5F19	−9.3	0.4	H‐bond	6	CYS A:47, CYS A:47, SER A:49, GLY A:135, GLY A:135, PRO A:154
Myricetin 3‐*O*‐hexuronide	NET	8hff	−9.8	0.9	H‐bond	9	ARG A:81, ARG A:81, LYS A:88, ASP A:310, ALA A:384, LYS A:541, LYS A:541, PRO A:542, THR A:544
Quercetin *O*‐glucuronide	NET	8hff	−9.7	1.1	H‐bond	7	TYR A:84, ALA A:384, ASP A:473, ASP A:473, THR A:544, ASP A:546, ASP A:546
Quercitrin	NET	8hff	−9.9	0.6	H‐bond	3	LYS A:88, ALA A:384, LYS A:541
Epicatechin	NET	8hff	−9.1	1.0	H‐bond	3	VAL A:148, PHE A:317, SER A:318
Myricitrin	NET	8hff	−10.3	0.6	H‐bond	7	ARG A:81, ARG A:81, LYS A:88, ASP A:310, ALA A:384, LYS A:541, LYS A:541
Myricetin	NET	8hff	−9.2	0.6	H‐bond	6	ARG A:81, ASP A:473, THR A:474, THR A:479, ASN A:539, ASN A:539
Hyperoside	NET	8hff	−9.8	0.3	H‐bond	6	ARG A:81, ARG A:81, THR A:306, ASP A:473, THR A:479, LYS A:541
Isoquercitrin	NET	8hff	−9.5	0.9	H‐bond	8	LYS A:88, THR A:306, ALA A:384, ASP A:473, ASP A:473, THR A:474, LYS A:541, LYS A:541
Quercetin	NET	8hff	−9.2	0.8	H‐bond	5	ARG A:81, ARG A:81, TYR A:84, SER A:536, SER A:536
Myricetin 3‐*O*‐pentoside	NET	8hff	−9.7	0.5	H‐bond	6	ARG A:81, ARG A:81, LYS A:88, THR A:313, ALA A:384, THR A:474
Myricetin 3‐*O*‐hexuronide	NOS2	2bhj	−9.9	0.3	H‐bond	8	ILE A:195, GLY A:196, GLY A:196, GLN A:199, SER A:236, TRP A:366, TRP A:366, TRP A:366
Myricetin 3‐*O*‐hexoside	NOS2	2bhj	−9.3	0.5	H‐bond	7	ARG A:193, GLY A:196, ALA A:237, ASN A:364, ASN A:364, TRP A:366, TYR A:483
Quercetin *O*‐glucuronide	NOS2	2bhj	−9.9	0.9	H‐bond	3	GLY A:196, GLN A:199, TYR A:483
Quercitrin	NOS2	2bhj	−10.0	0.9	H‐bond	3	GLN A:199, SER A:236, VAL A:346
Epicatechin	NOS2	2bhj	−9.1	0.7	H‐bond	4	GLY A:196, GLN A:199, TRP A:366, TRP A:366
Myricitrin	NOS2	2bhj	−9.2	1.0	H‐bond	5	GLN A:199, SER A:236, PRO A:344, VAL A:346, TRP A:366
Myricetin	NOS2	2bhj	−9.2	3.0	H‐bond	4	SER A:236, ASN A:364, TRP A:366, TRP A:366
3‐caffeoylquinic	NOS2	2bhj	−9.2	none	H‐bond	8	ILE A:195, GLY A:196, GLY A:196, GLY A:196, GLY A:196, TRP A:366, TRP A:366, TYR A:483
Hypericin	NOS2	2bhj	−11.0	1.0	H‐bond	5	GLN A:257, PRO A:344, ALA A:345, VAL A:346, TRP A:366
Hyperoside	NOS2	2bhj	−9.2	7.8	H‐bond	6	GLY A:196, GLN A:199, TRP A:366, TRP A:366, TRP A:366, TYR A:483
Isoquercitrin	NOS2	2bhj	−9.2	0.7	H‐bond	7	GLY A:196, GLN A:199, PRO A:344, TRP A:366, TRP A:366, TRP A:366, TYR A:483
Quercetin	NOS2	2bhj	−9.4	1.0	H‐bond	3	GLY A:196, GLN A:199, TYR A:483
Myricetin 3‐*O*‐pentoside	NOS2	2bhj	−9.2	0.8	H‐bond	6	TRP A:188, GLY A:196, GLY A:196, SER A:236, SER A:236, TRP A:366
Pseudohypericin	NOS2	2bhj	−10.6	0.4	H‐bond	7	GLN A:257, PRO A:344, ALA A:345, ALA A:345, VAL A:346, TRP A:366, TRP A:366
Quercetin *O*‐glucuronide	SERT	7mgw	−9.1	0.5	H‐bond	10	ASN A:211, THR A:221, SER A:224, ARG A:390, GLU A:392, ASP A:400, ALA A:401, GLU A:412, GLU A:412, PHE A:566
Hypericin	SERT	7mgw	−11.4	1.0	H‐bond	4	GLU A:229, GLU A:229, ASP A:400, GLN A:567
Pseudohypericin	SERT	7mgw	−10.7	0.0	H‐bond	2	THR A:221, GLN A:567

This study investigates the inhibitory potential of natural compounds on various target proteins through molecular docking analyses. Focusing on targets such as AChE, amylase, BChE, glucosidase, NET, NOS2, PTGS2, and SERT, this work provides a comprehensive comparison based on binding energies, hydrogen bonds, and interactions with critical amino acid residues. For the AChE target, compounds such as myricetin 3‐*O*‐hexuronide (10 hydrogen bonds, −10.8 kcal/mol), myricetin 3‐*O*‐hexoside (12 hydrogen bonds, −9.8 kcal/mol), quercetin *O*‐glucuronide (16 hydrogen bonds, −10.4 kcal/mol), quercitrin (11 hydrogen bonds, −10.1 kcal/mol), and hypericin (7 hydrogen bonds, −11.4 kcal/mol) showed strong inhibitory potential. Residues such as ASP A:74, TRP A:86, ASN A:87, GLY A:121, GLY A:122, GLU A:202, and TYR A:341 were frequently targeted, highlighting their importance in inhibitor binding. For the amylase target, compounds such as epicatechin (6 hydrogen bonds, −9.0 kcal/mol), myricitrin (11 hydrogen bonds, −9.3 kcal/mol), myricetin (5 hydrogen bonds, −9.3 kcal/mol), hypericin (5 hydrogen bonds, −10. 8 kcal/mol), and pseudohypericin (7 hydrogen bonds, −10.1 kcal/mol) showed significant interactions, with GLN A:63, ARG A:195, and ASP A:197 emerging as common binding sites. For the BChE target, myricetin 3‐*O*‐hexuronide (8 hydrogen bonds, −10.4 kcal/mol), quercitrin (12 hydrogen bonds, −10.4 kcal/mol), hypericin (5 hydrogen bonds, −12. 2 kcal/mol), and isoquercitrin (11 hydrogen bonds, −10.4 kcal/mol) showed high inhibitory potential, with TYR A:128, GLU A:197, ALA A:199, and HIS A:438 identified as key binding residues. For glucosidase, only hypericin (5 hydrogen bonds, −9.0 kcal/mol) showed a moderate interaction, suggesting a lower inhibitory potential for this target (Table 5; Figure 12). For the NET target, compounds such as myricetin 3‐*O*‐hexuronide (9 hydrogen bonds, −9.8 kcal/mol), quercetin *O*‐glucuronide (7 hydrogen bonds, −9.7 kcal/mol), myricitrin (7 hydrogen bonds, −10.3 kcal/mol), and isoquercitrin (8 hydrogen bonds, −9.5 kcal/mol) showed strong inhibitory potential, often interacting with residues ARG A:81, LYS A:88, and ALA A:384. For NOS2, myricetin 3‐*O*‐hexuronide (8 hydrogen bonds, −9.9 kcal/mol), epicatechin (4 hydrogen bonds, −9.1 kcal/mol), hypericin (5 hydrogen bonds, −11.0 kcal/mol), and pseudohypericin (7 hydrogen bonds, −10.6 kcal/mol) showed remarkable inhibitory properties, with residues GLY A:196, GLN A:199, and TRP A:366 being prominent. For PTGS2, myricetin 3‐*O*‐hexuronide (9 hydrogen bonds, −9.3 kcal/mol), myricitrin (7 hydrogen bonds, −9.2 kcal/mol) and pseudohypericin (6 hydrogen bonds, −9.3 kcal/mol) were notable, with ASN A:34, CYS A:47, and SER A:49 often acting as interaction sites. On the SERT target, quercetin *O*‐glucuronide (10 hydrogen bonds, −9.1 kcal/mol) and hypericin (4 hydrogen bonds, −11.4 kcal/mol) exhibited the strongest binding potential, specifically interacting with residues TYR A:124 and HIS A:447. In conclusion, compounds such as hypericin, pseudohypericin, quercetin *O*‐glucuronide, and myricitrin showed high binding affinity across multiple targets, engaging with a wide range of amino acid residues. Residues such as ASP A:74, TYR A:128, and GLY A:121 play a critical role in explaining the binding characteristics of these inhibitors with different targets. This analysis underscores the potential of these compounds as multi‐target inhibitors and highlights the importance of specific amino acid residues in mediating inhibitor interactions.

### 3.9 Binding Free Energy Analysis: MM/PBSA Results and Implications for Ligand Efficacy

**FIGURE 13:**
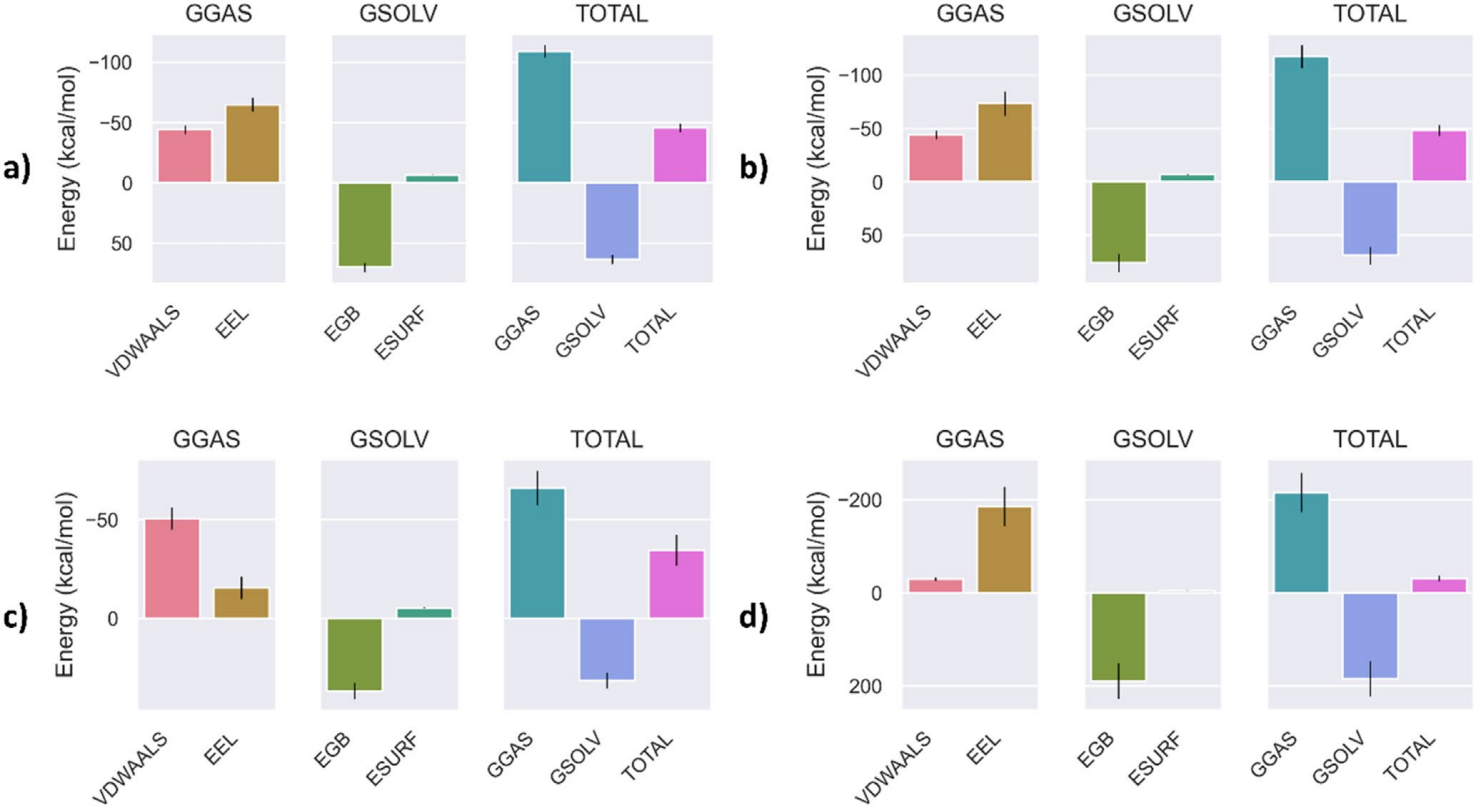
MM/PBSA binding free energy analysis: (a) NET_hyperoside complex, (b) NET_myricitrin complex, (c) NOS2_pseudohypericin complex, and (d) NOX2_myricetin‐3‐*O*‐hexuronide complex.

In this study, the effect of energy components on binding stability was evaluated by calculating and analyzing a series of protein–ligand complexes. The investigation focused on several important energy parameters, including van der Waals interaction (VDWAALS), electrostatic energy (EEL), polar solvation energy (EGB), surface tension (ESURF), gas phase energy (GGAS), solvation energy (GSOLV), and total energy (TOTAL). The evaluation of cancer‐related enzyme activities of compounds derived from *Hypericum* spp. was performed using MM/PBSA binding free energy calculations in conjunction with MD simulations. Five complexes were selected for further analysis based on factors such as low RMSD, high binding energy, and the number of hydrogen bonds formed. The complexes selected for further analysis are NET_hyperoside, NET_myricitrin, NOS2_pseudohypericin, and NOX2_myricetin‐3‐*O*‐hexuronide complexes shown in Table S3 (Figure 13).

The complexes selected for further analysis are as follows: The complexes with the lowest total energies are NET_hyperoside (total energy = −45.7 kcal/mol), NET_myricitrin (total energy = −48.3 kcal/mol), NOS2_pseudohypericin (total energy = −34.6 kcal/mol), and NOX2_myricetin‐3‐*O*‐hexuronide (total energy = −31.1 kcal/mol). These complexes were selected based on their favorable total energy values, stable binding profiles, and significant electrostatic and solvation energy contributions, which collectively indicate strong binding potential with their respective protein targets. The NET_hyperoside complex exhibited a notable total energy of −45.7 kcal/mol, supported by strong van der Waals interactions (VDWAALS = −44.2 kcal/mol) and electrostatic contributions (EEL = −64.8 kcal/mol). The solvation energy (GSOLV = 63.3 kcal/mol) balanced the interaction profile, indicating a favorable binding conformation and stability within the protein binding pocket. Similarly, the NET_myricitrin complex exhibited robust binding characteristics, with a total energy of −48.3 kcal/mol. The electrostatic interactions (EEL = −73.4 kcal/mol) were particularly prominent, indicating a strong electrostatic attraction between the ligand and protein. The van der Waals energy (VDWAALS = −44.0 kcal/mol) and solvation energy (GSOLV = 69.1 kcal/mol) contributed to an overall favorable interaction profile, thus making it a prime candidate for continued MD simulations. In the case of NOS2_pseudohypericin, the total energy was recorded at −34.6 kcal/mol. Although the van der Waals interactions (VDWAALS = −50.6 kcal/mol) were strong, the relatively weaker electrostatic contribution (EEL = −15.5 kcal/mol) and moderate solvation energy (GSOLV = 31.6 kcal/mol) suggest a balanced, albeit less potent, binding profile. This complex exhibited greater fluctuation in binding energy components, indicating a somewhat less stable interaction compared to the NET complexes. The NOX2_myricetin‐3‐*O*‐hexuronide complex, with a total energy of −31.1 kcal/mol, exhibited a distinctive energy profile characterized by robust electrostatic interactions (EEL = −185.7 kcal/mol) and a notable solvation energy (GSOLV = 184.5 kcal/mol). Notwithstanding the robust individual components, the overall binding energy was tempered by a comparatively diminished van der Waals contribution (VDWAALS = −29.9 kcal/mol). The observed stabilization following initial fluctuations suggests an adaptable, yet slightly less stable, binding conformation. In conclusion, the selected complexes—NET_hyperoside, NET_myricitrin, NOS2_pseudohypericin, and NOX2_myricetin‐3‐*O*‐hexuronide—demonstrate promising binding interactions, as evidenced by their favorable energy profiles. The strong van der Waals forces, balanced electrostatics, and appropriate solvation energies indicate that these compounds have the potential to act as effective inhibitors for the target proteins. While NOS2_pseudohypericin and NOX2_myricetin‐3‐*O*‐hexuronide display slightly more dynamic interaction profiles, NET_hyperoside and NET_myricitrin are distinguished by their stability and robust binding characteristics (Figure 13). These findings emphasize the necessity of evaluating the various energy components in order to determine the most promising candidates for continued MD studies. They suggest that *Hypericum* spp.–derived compounds have strong potential as therapeutic agents for targeting protein interactions related to cancer pathways.

### 3.10 Stability and Flexibility in MD Simulation

The objective of the study was to identify potential therapeutic drugs through a comprehensive investigation of the molecular interactions between specific ligands and target proteins, with a particular focus on elucidating their binding sites. Based on the results of the NET_hyperoside, NET_myricitrin, NOS2_pseudohypericin, and NOX2_myricetin‐3‐*O*‐hexuronide complexes, four complexes were selected for evaluation. These complexes demonstrated robust selectivity and stability in their interactions, as well as important criteria such as the presence of hydrogen‐bonding residues and the results of MM/PBSA binding free energy calculations. Subsequently, the complexes underwent MD simulations, which afforded greater insight into their biological efficacy and protein binding capacities, thus facilitating a more comprehensive evaluation of their potential as therapeutic agents.

**FIGURE 14:**
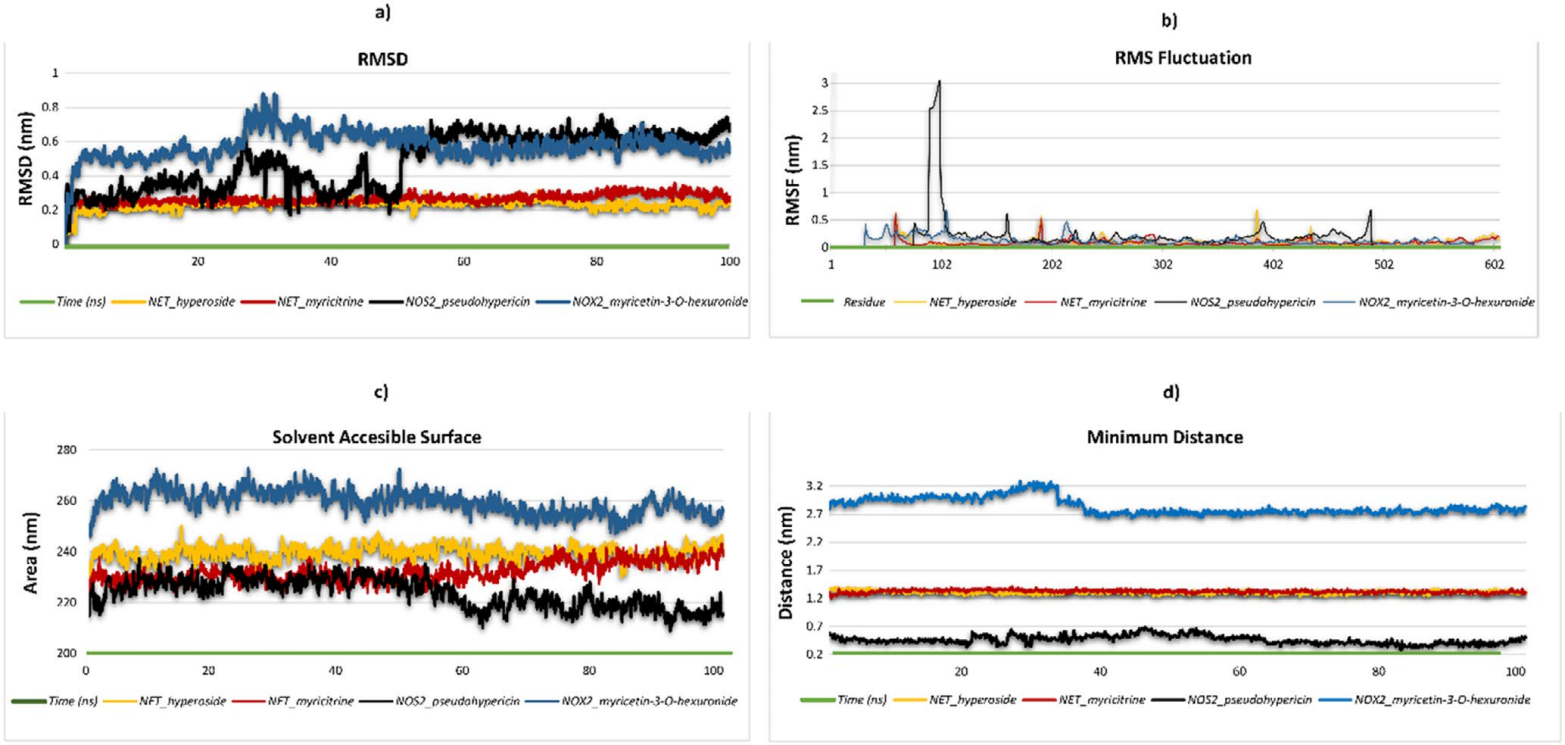
Presentation of molecular dynamics simulations in graphical form: (a) RMSD of NET_hyperoside, NET_myricitrin, NOS2_pseudohypericin, and NOX2_myricetin‐3‐*O*‐hexuronide complexes; (b) RMSF NET_hyperoside, NET_myricitrin, NOS2_pseudohypericin, and NOX2_myricetin‐3‐*O*‐hexuronide complexes; (c) solvent accessibility of NET_hyperoside, NET_myricitrin, NOS2_pseudohypericin, and NOX2_myricetin‐3‐*O*‐hexuronide complexes; and (d) minimum distance of NET_hyperoside, NET_myricitrin, NOS2_pseudohypericin, and NOX2_myricetin‐3‐*O*‐hexuronide.

The structural stability of four distinct ligand–protein complexes was evaluated through RMSD analysis, obtained from 100‐ns MD simulations. The NET_hyperoside complex displayed high structural stability, with RMSD values oscillating between 0.1 and 0.3 nm, indicative of robust interactions within the binding region. Similarly, the NET_myricitrin complex exhibited low RMSD values between 0.2 and 0.3 nm, indicating a stable conformational profile. In contrast, the NOS2_pseudohypericin complex exhibited higher structural variability, with RMSD fluctuations ranging from 0.2 to 0.7 nm during the initial 5 ns of the simulation. Thereafter, the RMSD values stabilized at 0.6–0.7 nm after 50 ns, indicating that the complex initially underwent conformational adjustments before reaching a relatively stable structure. The NOX2_myricetin‐3‐*O*‐hexuronide complex exhibited the greatest RMSD fluctuations among the complexes under analysis, with extensive variations observed up to 60 ns, after which the RMSD values stabilized. In general, the NET_hyperoside and NET_myricitrin complexes demonstrated the most stable conformational profiles, exhibiting low and consistent RMSD values, which indicate stronger binding interactions. In contrast, the NOS2_pseudohypericin and NOX2_myricetin‐3‐*O*‐hexuronide complexes exhibited higher structural flexibility and lower stability. These findings suggest that the binding regions of the NET_hyperoside and NET_myricitrin complexes are better configured and more stable, thereby highlighting the positive impact of strong binding interactions on structural stability (Figure 14a). The RMSF plot is a useful tool for visualizing the flexibility levels and adaptability of residues in protein complexes. The NET_hyperoside and NET_myricitrin complexes exhibit low RMSF values across the residues, with fluctuations generally remaining below 0.5 nm. This indicates that the residues within the binding region exhibit limited mobility, thereby suggesting a stable structural conformation. In contrast, the NOS2_pseudohypericin complex displays a notable increase in flexibility, particularly around residue 102, where the RMSF value reaches up to 3 nm. These fluctuations occur in the loop regions of the protein, indicating increased mobility and dynamic changes in areas not directly involved in the binding site. Similarly, a notable increase in flexibility is observed around residue 502, which may indicate the occurrence of localized structural changes. The NOX2_myricetin‐3‐*O*‐hexuronide complex generally exhibits low root‐mean‐square fluctuation (RMSF) values, although slight increases are observed around residues 102 and 402. These increases are not located in the binding region, yet they suggest localized flexibility and potential conformational mobility. In conclusion, the NET_hyperoside, NET_myricitrin, and NOS2_pseudohypericin complexes display the most stable and rigid structures, as evidenced by their low RMSF values and reduced flexibility in the binding regions. In contrast, the NOX2_myricetin‐3‐*O*‐hexuronide complex displays higher RMSF fluctuations around specific residues, indicating greater structural flexibility and dynamic motion. This suggests that loop and surface regions outside the binding site significantly contribute to the overall structural flexibility (Figure 14b). The solvent exposure levels of the ligand–protein complexes were evaluated utilizing the solvent accessible surface area (SASA) method. The findings demonstrate that the NOX2_myricetin‐3‐*O*‐hexuronide complex exhibits the highest SASA values, with an average of approximately 260 nm^2^. This elevated SASA value indicates that the complex possesses a larger surface area and is more extensively exposed to solvent interactions. In contrast, the NOS2_pseudohypericin complex exhibits the lowest average SASA value, approximately 220 nm^2^, indicating that it has a more compact structure with limited solvent exposure. The NET_hyperoside and NET_myricitrin complexes exhibit intermediate SASA values, ranging between 240 and 245 nm^2^, which suggest moderate solvent exposure. These values suggest that both complexes have relatively compact structures and limited interactions with the solvent. The SASA profile of the NET_myricitrin complex exhibits similarities to that of NET_hyperoside, although with slight fluctuations. In general, the elevated SASA values observed for the NOX2_myricetin‐3‐*O*‐hexuronide complex reflect a larger surface area and increased solvent interaction, whereas the reduced SASA values of the NOS2_pseudohypericin complex indicate a more compact and solvent‐shielded structure. These findings underscore the potential influence of surface area and solvent interactions on the structural stability and dynamics of the complexes (Figure 14c). In the course of the 100‐ns simulation, the minimum distance measurements of the ligand–protein complexes were subjected to analysis. The NOX2_myricetin‐3‐*O*‐hexuronide complex exhibited the highest minimum distance profile among the complexes under study, with values ranging from 2.7 to 3.2 nm. This complex exhibited considerable fluctuations during the initial 40 ns of the simulation period, but subsequently attained a more stable configuration. The minimum distance profiles of the NET_hyperoside and NET_myricitrin complexes were found to be similar, with both stabilizing within the range of 1.2–1.3 nm. Both complexes exhibited minimal fluctuations, indicating that the interactions within the binding region were sustained and stable. In contrast, the NOS2_pseudohypericin complex exhibited lower minimum distance values, with fluctuations between 0.3 and 0.7 nm. The minor fluctuations observed during the initial 60 ns period may be indicative of an adaptation phase within the binding region, followed by a more stable distance profile after this period. In general, the higher minimum distance values observed for the NOX2_myricetin‐3‐*O*‐hexuronide complex suggest a more expansive spatial configuration within the binding region and a more flexible binding conformation. In contrast, the NET_hyperoside, NET_myricitrin, and NOS2_pseudohypericin complexes exhibited lower and more consistent minimum distance values, indicative of tighter binding interactions. These findings emphasize the dynamic nature of the binding interactions and the alterations in stability over time within the binding regions of these complexes (Figure 14d).

**FIGURE 15:**
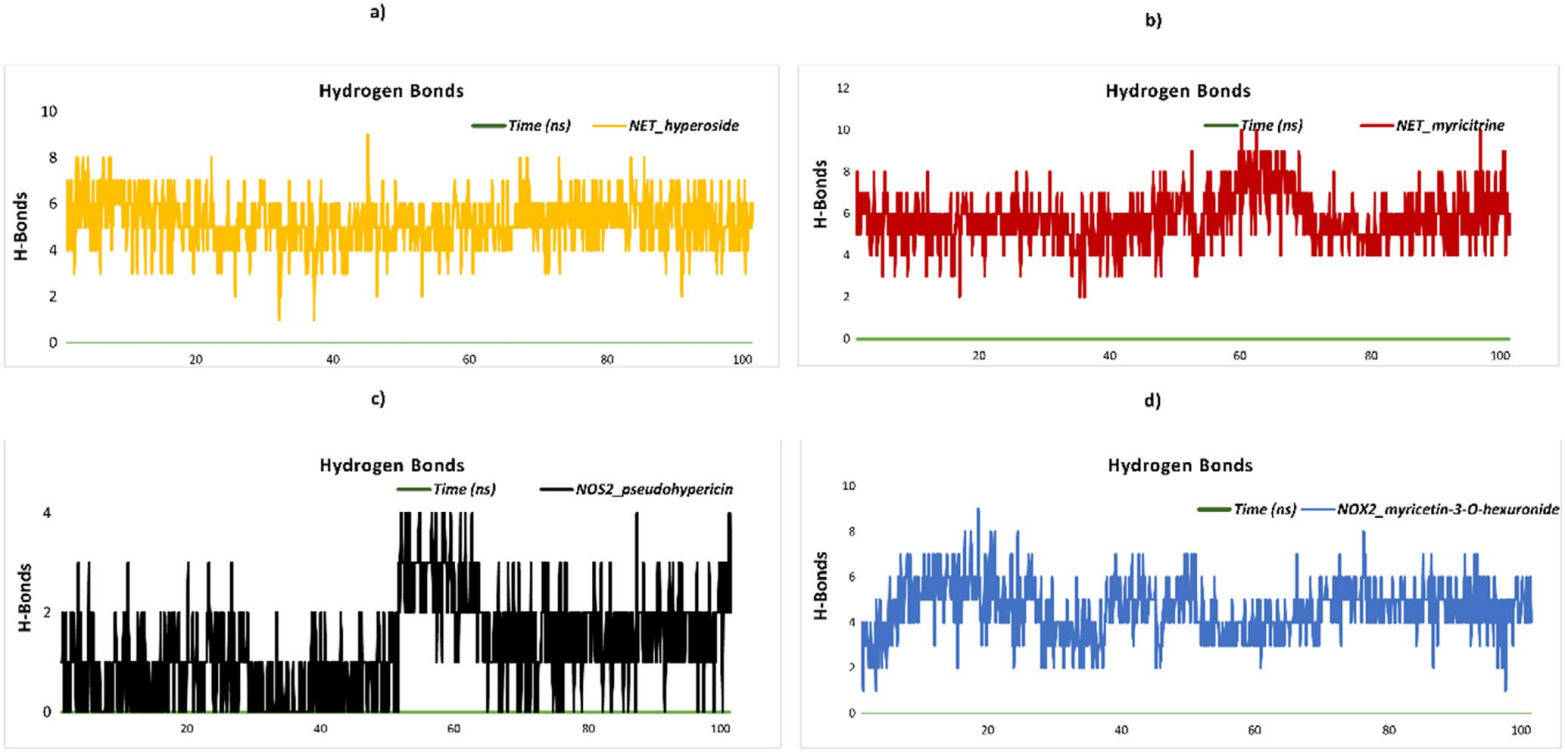
Hydrogen bond analysis of ligand–protein complexes over a 100‐ns simulation period: (a) hydrogen bonds in NET_hyperoside complex, (b) hydrogen bonds in the NET_myricitrin complex, (c) hydrogen bonds in the NOS2_pseudohypericin complex, and (d) hydrogen bonds in the NOX2_myricetin‐3‐*O*‐hexuronide complex.

The hydrogen bond dynamics of four ligand–protein complexes were analyzed over the course of a 100‐ns MD simulation. In the NET_hyperoside complex (Figure 15a), the number of hydrogen bonds exhibited fluctuations between 1 and 9 throughout the simulation. During the initial 20 ns, considerable fluctuations were observed, with the hydrogen bond count typically oscillating between 4 and 8. From 20 to 60 ns, a more stable pattern emerged, with the majority of hydrogen bonds stabilizing between 5 and 7. In the final phase of the simulation (60–100 ns), the hydrogen bond count remained generally between 4 and 6, indicating the formation of a structurally stable hydrogen bond network in the binding region. In the NET_myricitrin complex (Figure 15b), the number of hydrogen bonds exhibited variability between 2 and 10. In contrast to the initial variability observed in the preceding complex, the hydrogen bond count remained relatively stable during the initial 20 ns, fluctuating between 2 and 8. Between 20 and 50 ns, the hydrogen bond count demonstrated reduced consistency, oscillating between 5 and 8. Nevertheless, following a period of 50 ns, the hydrogen bonds exhibited a tendency toward stability, with a consistent count observed for the remainder of the simulation. With regard to the NOS2_pseudohypericin complex (Figure 15c), it was observed that the hydrogen bond count was consistently lower, fluctuating mainly between 0 and 4, with a predominant range of 0–1 during the majority of the simulation. In the interval between 60 and 100 ns, the number of hydrogen bonds ranged from 1 to 3, with a tendency toward two hydrogen bonds. However, the pattern lacked stability, indicating weaker and less consistent binding interactions. The NOX2_myricetin‐3‐*O*‐hexuronide complex (Figure 15d) displayed a more extensive range of hydrogen bond fluctuations, with values spanning between 1 and 9. During the initial 20 ns, considerable variability was evident, with the hydrogen bond count frequently reaching values between 8 and 10. From 20 to 60 ns, the count exhibited a period of stability, with a predominant range of 4–5. Following a period of 70 ns, the hydrogen bond count exhibited a further stabilization, indicative of the establishment of a more consistent binding interaction in the later stages of the simulation.

In conclusion, the results of the MD simulations and hydrogen bond analyses indicate that the NET_hyperoside and NET_myricitrin complexes demonstrate relatively stable binding interactions, characterized by consistent hydrogen bonding and compact structural conformations. These complexes demonstrate robust binding interactions, supported by a stable hydrogen bond network. In contrast, the NOS2_pseudohypericin complex displays weaker and more inconsistent hydrogen bond patterns, which reflect less stable binding interactions. Despite initial variability, the NOX2_myricetin‐3‐*O*‐hexuronide complex achieved stabilization after 70 ns, indicating a gradual adaptation process in the binding region. In conclusion, the stable hydrogen‐bonding profiles of NET_hyperoside and NET_myricitrin indicate their potential as strong therapeutic candidates, whereas the variability observed in NOS2_pseudohypericin suggests a less stable binding conformation.

## 4 Conclusion

In conclusion, the present study investigated the chemical composition and the biopharmacological potential of ethanol/water (70%) and water extracts from three *Hypericum* species, namely *H. scabrum* , *H. lysimachioides*, and *H. uniglandulosum*. In particular, the study demonstrated antioxidant and anti‐inflammatory effects that were more prominent for *H. scabrum* and *H. lysimachioides*, which also displayed the highest content in phenolic and flavonoid compounds. By contrast, *H. uniglandulosum* extracts exerted a more potent neuromodulatory role on serotonin and norepinephrine release through the inhibition of SERT and NET gene expression, respectively, which poses the basis for future exploration of the antidepressant effect in in vivo models of depression. Bioinformatic analyses revealed enrichment in pathways related to neurodegeneration and mood disorders, identifying key targets such as IL‐6, MAPK, and NOS2. Molecular docking further validated these findings, showing strong binding affinities of key phytochemicals to target proteins, while MD simulations confirmed the stability of ligand–protein interactions, especially for NET_hyperoside and NET_myricitrin complexes. Further studies are still needed for elucidating the mechanisms underlying the inhibition of monoamine transporters exerted by the present extracts.

## Author Contributions

**Muammer Bahsi:** data curation (equal), formal analysis (equal), funding acquisition (equal), investigation (lead), methodology (equal), project administration (equal), software (equal), supervision (equal), validation (equal), visualization (equal), writing – original draft (equal). **Simonetta Cristina Di Simone:** conceptualization (equal), data curation (equal), formal analysis (equal), funding acquisition (equal), investigation (lead), methodology (equal), project administration (equal), resources (equal), software (equal), supervision (equal), validation (equal), visualization (equal), writing – original draft (equal). **Dimitrina Zheleva‐Dimitrova:** conceptualization (equal), data curation (equal), formal analysis (equal), funding acquisition (equal), investigation (equal), methodology (equal), project administration (equal), resources (equal), software (equal), supervision (equal), validation (equal), visualization (equal), writing – original draft (equal). **Gokhan Zengin:** conceptualization (equal), data curation (equal), formal analysis (equal), funding acquisition (equal), investigation (equal), methodology (equal), project administration (equal), resources (equal), software (equal), supervision (equal), validation (equal), visualization (equal), writing – original draft (equal). **Gaia Cusumano:** conceptualization (equal), data curation (equal), formal analysis (equal), funding acquisition (equal), investigation (equal), methodology (equal), project administration (equal), resources (equal), software (equal), supervision (equal), validation (equal), visualization (equal). **Giancarlo Angeles Flores:** conceptualization (equal), data curation (equal), formal analysis (equal), funding acquisition (equal), investigation (equal), methodology (equal), project administration (equal), resources (equal), software (equal), supervision (equal), validation (equal), visualization (equal). **Paola Angelini:** conceptualization (equal), data curation (equal), formal analysis (equal), funding acquisition (equal), investigation (equal), methodology (equal), project administration (equal), resources (equal), software (equal), supervision (equal), validation (equal), visualization (equal). **Carla Emiliani:** conceptualization (equal), data curation (equal), formal analysis (equal), funding acquisition (equal), investigation (equal), methodology (equal), project administration (equal), resources (equal), software (equal), supervision (equal), validation (equal), visualization (equal). **Mehmet Veysi Cetiz:** conceptualization (equal), data curation (equal), formal analysis (equal), funding acquisition (equal), investigation (equal), methodology (equal), project administration (equal), resources (equal), software (equal), supervision (equal), validation (equal), visualization (equal). **Annalisa Chiavaroli:** conceptualization (equal), data curation (equal), formal analysis (equal), funding acquisition (equal), investigation (equal), methodology (equal), project administration (equal), resources (equal), software (equal), supervision (equal), validation (equal), visualization (equal). **Luigi Menghini:** conceptualization (equal), data curation (equal), formal analysis (equal), funding acquisition (equal), methodology (equal), project administration (equal), resources (equal), software (equal), supervision (equal), validation (equal), visualization (equal). **Guistino Orlando:** conceptualization (equal), data curation (equal), formal analysis (equal), funding acquisition (equal), investigation (equal), methodology (equal), project administration (equal), resources (equal), software (equal), supervision (equal), validation (equal), visualization (equal). **Claudio Ferrante:** conceptualization (equal), data curation (equal), formal analysis (equal), funding acquisition (equal), investigation (lead), methodology (equal), project administration (equal), resources (equal), software (equal), supervision (equal), validation (equal), visualization (equal), writing – original draft (equal), writing – review and editing (lead).

## Consent

The authors have nothing to report.

## Conflicts of Interest

The authors declare no conflicts of interest.

## Supporting information


Data S1.

## Data Availability

Data will be made available upon request.
