# Supplementary material for: Unlocking New Pharma/Nutraceutical Frontiers With Neuroprotective Properties of Three Hypericum Species: A Study Combination With In Vitro and In Silico Methodologies
Source: Food Sci Nutr. 2025 Apr 10;13(4):e70069. doi: 10.1002/fsn3.70069 (PMC11983157; doi:10.1002/fsn3.70069)
Supplement: Supplementary file 1 — Data S1. [file FSN3-13-e70069-s001.docx]

**Unlocking new pharma/nutraceutical frontiers with neuroprotective properties of three *Hypericum* species: A study combination with *invitro* and *in silico* methodologies.**

Muammer Bahsi^1#^, Simonetta Cristina Di Simone^2#^, Dimitrina Zheleva-Dimitrova^3^, Gokhan Zengin^4,^ Gaia Cusumano^5^, Giancarlo Angeles Flores^2,5^, Paola Angelini^5^, Carla Emiliani^5^, Mehmet Veysi Cetiz^6^, Annalisa Chiavaroli^2^, Luigi Menghini^2^, Guistino Orlando^2^, Claudio Ferrante^2*^

*^1^Firat University, Faculty of Education, 23000, Elazığ, Turkey*, [muammerbahsi@firat.edu.tr](mailto:muammerbahsi@firat.edu.tr) (MB)

*^2^Botanic Garden “Giardino dei Semplici”, Department of Pharmacy, “Gabriele d'Annunzio” University, Chieti, Italy*

*^3^Department of Pharmacognosy, Faculty of Pharmacy, Medical University of Sofia, 1000 Sofia, Bulgaria*, [dzheleva@pharmfac.mu-sofia.bg](mailto:dzheleva@pharmfac.mu-sofia.bg) (DZ)

*^4^Department of Biology, Science Faculty, Selcuk University, Konya, Turkey*, [gokhanzengin@selcuk.edu.tr](mailto:gokhanzengin@selcuk.edu.tr) (GZ)

*^5^Department of Chemistry, Biology and Biotechnology, University of Perugia, Perugia, Italy*

*^6^Department of Medical Biochemistry, Faculty of Medicine, Harran University, Sanliurfa, 63290, Turkey*, [mvcetiz@gmail.com](mailto:mvcetiz@gmail.com) (MVC)

^#^The authors equally contributed.

Corresponding author: claudio.ferrante@unich.it

**Supplementary Tables**

**Table S1.** Relevant protein and enzyme target coordinates of the docking box.

| **Group** | ***Target*** | **PDB ID** | **Grid size X, Y, Z** | **X, Y, Z dimensions** | **Reference** |
| --- | --- | --- | --- | --- | --- |
| ***Enzymes*** | *AChE* | 2y2v | 22 Å X 30 Å X 40 Å | 31.062, 20.311, 11.947 | (Cusumano et al., 2024} |
|  | *BChE* | 3djy | 30 Å X 30 Å X 30 Å | 44.794, -19.63, -25.227 | (Duran et al., 2024) |
|  | *Tyr* | 5m8o | 26 Å X 26 Å X 28 Å | -13.194, 5.341, -26.28 | (Yagi et al., 2024;Kurt‐Celep et al., 2024) |
|  | *Amylase* | 2qv4 | 28 Å 28 Å X 24 Å | 14.188, 48.964, 22.886 | (Zengin et al., 2024) |
|  | *Glucosidase* | 3w37 | 42 Å 52 Å X 54 Å | 3.091, −8.008, −4.08 | (Cetiz et al., 2024) |
| **Disease-related proteins** | *COX2* | 3ln1 | 40 Å X 52 Å X 40 Å | 1.766, 19.665, 32.654 | Center of inhibitor |
|  | *NOS2* | 2bhj | 40 Å X 40 Å X 40 Å | 24.854, 42.425, -12.575 | Center of inhibitor |
|  | *SERT* | 7mgw | 64 Å X 34 Å X 28 Å | 25.126,1.027, 51.326 | Center of inhibitor |
|  | *NET* | *8hff* | 40 Å X 112 Å X 40 Å | 26.657, 17.779, 12.31 | Center of inhibitor |
|  | *IL1B* | 1itb | 30 Å 28 Å X 28 Å | 42.61, 16.62, 32.82 | (Yu et al., 2010) |
|  | *IL6* | 5fuc | 40 Å X 40 Å X 40 Å | -5.632, -11.8, 21.568 | Center of inhibitor |
|  | *PTGS2* | 5f19 | 40 Å X 54 Å X 76 Å | -2.96, -15.097, 46.845 | (Yu et al., 2010) |

**Table S2.** Relevant protein and enzyme result of the docking scores

| **Group** | **Compound** | **PDB ID** | **Binding energy** | **PDB ID** | **Binding energy** | **PDB ID** | **Binding energy** | **PDB ID** | **Binding energy** | **PDB ID** | **Binding energy** |
| --- | --- | --- | --- | --- | --- | --- | --- | --- | --- | --- | --- |
| **Enzyme** | Myricetin 3-O-hexuronide | 2qv4 | -8.2 | 3w37 | -8.5 | 2y2v | -10.8 | 3djy | -10.4 | 5m8o | -7.7 |
|  | Myricetin 3-O-hexoside | 2qv4 | -8.9 | 3w37 | -7.9 | 2y2v | -9.8 | 3djy | -9.9 | 5m8o | -7.6 |
|  | Quercetin O-glucuronide | 2qv4 | -8.9 | 3w37 | -8.4 | 2y2v | -10.4 | 3djy | -10.2 | 5m8o | -8.0 |
|  | Quercitrin | 2qv4 | -8.9 | 3w37 | -7.9 | 2y2v | -10.1 | 3djy | -10.4 | 5m8o | -7.7 |
|  | Epicatechin | 2qv4 | -9.0 | 3w37 | -8.3 | 2y2v | -9.6 | 3djy | -9.2 | 5m8o | -7.7 |
|  | Myricitrin | 2qv4 | -9.3 | 3w37 | -8.1 | 2y2v | -10.2 | 3djy | -10.4 | 5m8o | -7.9 |
|  | Myricetin | 2qv4 | -9.3 | 3w37 | -8.5 | 2y2v | -10.2 | 3djy | -9.3 | 5m8o | -7.2 |
|  | 3-O-caffeoylquinic acid | 2qv4 | -8.2 | 3w37 | -8.0 | 2y2v | -9.4 | 3djy | -8.2 | 5m8o | -7.6 |
|  | 5-O-caffeoylquinic acid | 2qv4 | -8.1 | 3w37 | -7.7 | 2y2v | -9.3 | 3djy | -8.1 | 5m8o | -8.2 |
|  | Hypericin | 2qv4 | -10.8 | 3w37 | -9.0 | 2y2v | -11.4 | 3djy | -12.2 | 5m8o | -7.9 |
|  | 4-p-coumaroylquinic acid | 2qv4 | -8.0 | 3w37 | -7.6 | 2y2v | -9.2 | 3djy | -8.9 | 5m8o | -6.8 |
|  | Hyperoside | 2qv4 | -8.8 | 3w37 | -7.9 | 2y2v | -10.0 | 3djy | -10.4 | 5m8o | -7.3 |
|  | Isoquercitrin | 2qv4 | -8.4 | 3w37 | -8.0 | 2y2v | -8.6 | 3djy | -10.4 | 5m8o | -7.1 |
|  | 4-O-caffeoylquinic acid | 2qv4 | -7.7 | 3w37 | -7.8 | 2y2v | -9.2 | 3djy | -8.1 | 5m8o | -7.3 |
|  | Quercetin | 2qv4 | -9.2 | 3w37 | -8.1 | 2y2v | -9.5 | 3djy | -9.4 | 5m8o | -7.9 |
|  | Isocitric acid | 2qv4 | -6.3 | 3w37 | -6.2 | 2y2v | -6.4 | 3djy | -6.0 | 5m8o | -6.7 |
|  | Myricetin 3-O-pentoside | 2qv4 | -9.0 | 3w37 | -8.7 | 2y2v | -10.5 | 3djy | -9.5 | 5m8o | -7.5 |
|  | 5-p-coumaroylquinic acid | 2qv4 | -8.3 | 3w37 | -7.6 | 2y2v | -9.5 | 3djy | -8.1 | 5m8o | -7.7 |
|  | Pseudohypericin | 2qv4 | -10.1 | 3w37 | -8.7 | 2y2v | -10.5 | 3djy | -11.6 | 5m8o | -7.4 |
| **Neuroinflammation** | Myricetin 3-O-hexuronide | 1itb | -7.3 | 5fuc | -7.1 | 5f19 | -9.3 |  |  |  |  |
|  | Myricetin 3-O-hexoside | 1itb | -7.1 | 5fuc | -6.4 | 5f19 | -8.8 |  |  |  |  |
|  | Quercetin O-glucuronide | 1itb | -6.8 | 5fuc | -7.4 | 5f19 | -8.9 |  |  |  |  |
|  | Quercitrin | 1itb | -7.3 | 5fuc | -7.2 | 5f19 | -8.8 |  |  |  |  |
|  | Epicatechin | 1itb | -6.4 | 5fuc | -6.8 | 5f19 | -9.0 |  |  |  |  |
|  | Myricitrin | 1itb | -7.2 | 5fuc | -7.1 | 5f19 | -9.2 |  |  |  |  |
|  | Myricetin | 1itb | -6.8 | 5fuc | -6.6 | 5f19 | -9.1 |  |  |  |  |
|  | 3-O-caffeoylquinic acid | 1itb | -6.5 | 5fuc | -6.9 | 5f19 | -7.7 |  |  |  |  |
|  | 5-O-caffeoylquinic acid | 1itb | -6.8 | 5fuc | -6.8 | 5f19 | -7.9 |  |  |  |  |
|  | Hypericin | 1itb | -8.7 | 5fuc | -8.0 | 5f19 | -9.9 |  |  |  |  |
|  | 4-p-coumaroylquinic acid | 1itb | -6.6 | 5fuc | -6.3 | 5f19 | -7.8 |  |  |  |  |
|  | Hyperoside | 1itb | -6.9 | 5fuc | -7.1 | 5f19 | -8.9 |  |  |  |  |
|  | Isoquercitrin | 1itb | -7.0 | 5fuc | -7.2 | 5f19 | -8.9 |  |  |  |  |
|  | 4-O-caffeoylquinic acid | 1itb | -7.0 | 5fuc | -6.8 | 5f19 | -8.3 |  |  |  |  |
|  | Quercetin | 1itb | -6.6 | 5fuc | -6.6 | 5f19 | -9.5 |  |  |  |  |
|  | Isocitric acid | 1itb | -5.3 | 5fuc | -5.4 | 5f19 | -6.7 |  |  |  |  |
|  | Myricetin 3-O-pentoside | 1itb | -7.3 | 5fuc | -6.9 | 5f19 | -8.9 |  |  |  |  |
|  | 5-p-coumaroylquinic acid | 1itb | -6.4 | 5fuc | -6.6 | 5f19 | -7.5 |  |  |  |  |
|  | Pseudohypericin | 1itb | -8.4 | 5fuc | -7.7 | 5f19 | -9.3 |  |  |  |  |
| **Neuromodulator** | Myricetin 3-O-hexuronide | 5f19 | -9.3 | 8hff | -9.8 | 2bhj | -9.9 | 7mgw | -8.6 | 5f19 | -9.3 |
|  | Myricetin 3-O-hexoside | 5f19 | -8.8 | 8hff | -8.9 | 2bhj | -9.3 | 7mgw | -8.2 | 5f19 | -8.8 |
|  | Quercetin O-glucuronide | 5f19 | -8.9 | 8hff | -9.7 | 2bhj | -9.9 | 7mgw | -9.1 | 5f19 | -8.9 |
|  | Quercitrin | 5f19 | -8.8 | 8hff | -9.9 | 2bhj | -10.0 | 7mgw | -8.7 | 5f19 | -8.8 |
|  | Epicatechin | 5f19 | -9.0 | 8hff | -9.1 | 2bhj | -9.1 | 7mgw | -8.6 | 5f19 | -9.0 |
|  | Myricitrin | 5f19 | -9.2 | 8hff | -10.3 | 2bhj | -9.2 | 7mgw | -8.6 | 5f19 | -9.2 |
|  | Myricetin | 5f19 | -9.1 | 8hff | -9.2 | 2bhj | -9.2 | 7mgw | -7.4 | 5f19 | -9.1 |
|  | 3-O-caffeoylquinic acid | 5f19 | -7.7 | 8hff | -8.8 | 2bhj | -9.2 | 7mgw | -7.6 | 5f19 | -7.7 |
|  | 5-O-caffeoylquinic acid | 5f19 | -7.9 | 8hff | -8.9 | 2bhj | -8.4 | 7mgw | -7.5 | 5f19 | -7.9 |
|  | Hypericin | 5f19 | -9.9 | 8hff | -8.4 | 2bhj | -11.0 | 7mgw | -11.4 | 5f19 | -9.9 |
|  | 4-p-coumaroylquinic acid | 5f19 | -7.8 | 8hff | -8.4 | 2bhj | -8.3 | 7mgw | -8.0 | 5f19 | -7.8 |
|  | Hyperoside | 5f19 | -8.9 | 8hff | -9.8 | 2bhj | -9.2 | 7mgw | -8.1 | 5f19 | -8.9 |
|  | Isoquercitrin | 5f19 | -8.9 | 8hff | -9.5 | 2bhj | -9.2 | 7mgw | -8.1 | 5f19 | -8.9 |
|  | 4-O-caffeoylquinic acid | 5f19 | -8.3 | 8hff | -8.6 | 2bhj | -8.3 | 7mgw | -8.1 | 5f19 | -8.3 |
|  | Quercetin | 5f19 | -9.5 | 8hff | -9.2 | 2bhj | -9.4 | 7mgw | -8.5 | 5f19 | -9.5 |
|  | Isocitric acid | 5f19 | -6.7 | 8hff | -6.2 | 2bhj | -5.8 | 7mgw | -6.3 | 5f19 | -6.7 |
|  | Myricetin 3-O-pentoside | 5f19 | -8.9 | 8hff | -9.7 | 2bhj | -9.2 | 7mgw | -8.4 | 5f19 | -8.9 |
|  | 5-p-coumaroylquinic acid | 5f19 | -7.5 | 8hff | -8.6 | 2bhj | -8.4 | 7mgw | -7.4 | 5f19 | -7.5 |
|  | Pseudohypericin | 5f19 | -9.3 | 8hff | -8.1 | 2bhj | -10.6 | 7mgw | -10.7 | 5f19 | -9.3 |

**Table S3.** Selected protein-ligand complexes for MM/PBSA binding free energy analysis based on molecular dynamics simulations.

| **Complex** | **Frames** | **VDWAALS** | **EEL** | **EGB** | **ESURF** | **GGAS** | **GSOLV** | **TOTAL** |
| --- | --- | --- | --- | --- | --- | --- | --- | --- |
| NET_hyperoside | Average | -44.21 | -64.75 | 69.92 | -6.64 | -108.96 | 63.28 | -45.68 |
|  | SD | 3.52 | 5.84 | 3.85 | 0.18 | 5.16 | 3.77 | 3.68 |
|  | SEM | 0.12 | 0.19 | 0.13 | 0.01 | 0.17 | 0.13 | 0.12 |
| NET_myricitrine | Average | -44 | -73.38 | 75.99 | -6.89 | -117.38 | 69.1 | -48.28 |
|  | SD | 3.91 | 11.52 | 8.59 | 0.33 | 10.88 | 8.39 | 5.1 |
|  | SEM | 0.13 | 0.38 | 0.29 | 0.01 | 0.36 | 0.28 | 0.17 |
| NOS2_hypericin | Average | -43.34 | -12.78 | 35.18 | -5.56 | -56.11 | 29.62 | -26.49 |
|  | SD | 2.59 | 9.05 | 6.85 | 0.29 | 9.72 | 6.81 | 4.26 |
|  | SEM | 0.09 | 0.3 | 0.23 | 0.01 | 0.32 | 0.23 | 0.14 |
| NOS2_pseudohypericin | Average | -50.57 | -15.53 | 36.89 | -5.34 | -66.1 | 31.55 | -34.55 |
|  | SD | 5.64 | 5.67 | 4.12 | 0.3 | 8.71 | 4.03 | 7.78 |
|  | SEM | 0.2 | 0.2 | 0.15 | 0.01 | 0.31 | 0.14 | 0.27 |
| NOX2_myricetin-3-O-hexuronide | Average | -29.94 | -185.66 | 189.6 | -5.13 | -215.6 | 184.47 | -31.13 |
|  | SD | 3.75 | 42.23 | 38.32 | 0.5 | 42.12 | 37.95 | 6.06 |
|  | SEM | 0.17 | 1.89 | 1.71 | 0.02 | 1.88 | 1.7 | 0.27 |
| SERT_hypericin | Average | -31.99 | -9.84 | 26.66 | -3.7 | -41.82 | 22.96 | -18.87 |
|  | SD | 5.65 | 8.94 | 8.76 | 0.65 | 10.2 | 8.6 | 4.77 |
|  | SEM | 0.25 | 0.4 | 0.39 | 0.03 | 0.46 | 0.38 | 0.21 |
